# Single-Stranded DNA Aptamers against Pathogens and Toxins: Identification and Biosensing Applications

**DOI:** 10.1155/2015/419318

**Published:** 2015-06-23

**Authors:** Ka Lok Hong, Letha J. Sooter

**Affiliations:** Department of Basic Pharmaceutical Sciences, 1 Medical Center Drive, P.O. Box 9530, Morgantown, WV 20506, USA

## Abstract

Molecular recognition elements (MREs) can be short sequences of single-stranded DNA, RNA, small peptides, or antibody fragments. They can bind to user-defined targets with high affinity and specificity. There has been an increasing interest in the identification and application of nucleic acid molecular recognition elements, commonly known as aptamers, since they were first described in 1990 by the Gold and Szostak laboratories. A large number of target specific nucleic acids MREs and their applications are currently in the literature. This review first describes the general methodologies used in identifying single-stranded DNA (ssDNA) aptamers. It then summarizes advancements in the identification and biosensing application of ssDNA aptamers specific for bacteria, viruses, their associated molecules, and selected chemical toxins. Lastly, an overview of the basic principles of ssDNA aptamer-based biosensors is discussed.

## 1. Introduction

Target detection in diagnostics and sensors relies on successful molecular recognitions. Traditionally, antibodies have been used in biosening applications due to their target specificities and affinities. However, the inherent properties of proteins give rise to many shortcomings of antibodies. In 1990, the Gold Laboratory first described a process, termed Systematic Evolution of Ligands by Exponential Enrichment (SELEX) [1], which identifies one or few molecular recognition elements (MREs) with high affinity and specificity toward their intended targets. MREs can be short sequences of single-stranded DNA, RNA, small peptides, or antibody fragments. All types of MREs are capable of binding to user-defined targets with high affinity and specificity, and these targets include proteins, small molecules, viruses, whole bacteria cells, and mammalian cells [2].

**Figure 1:**
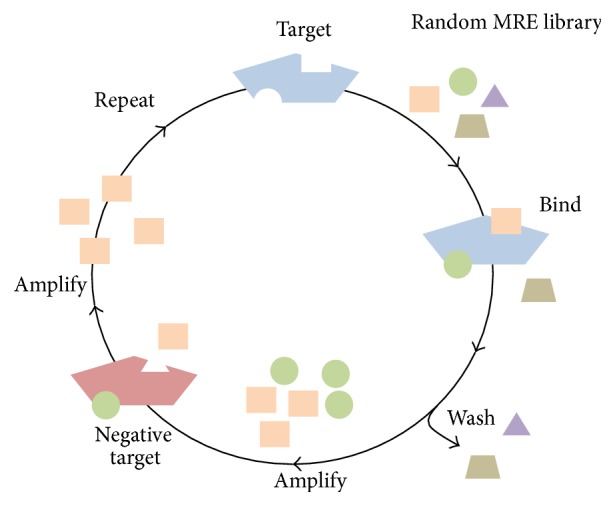
Illustration of the SELEX process. A random library consisting of up to 10^15^ single-stranded nucleic acids molecules are incubated with the target of interest. Those that bound to the target are retrieved and amplified by polymerase case reaction. It is then followed by incubation with negative targets. Those that do not bind to negative targets are retained, amplified, and subjected to further rounds of* in vitro* selection.

In order to identify nucleic acid MREs, the SELEX process generally begins from a very large random library consisting of 10^13^ to 10^15^ different molecules. An individual nucleic acid MRE is composed of two constant regions for primer attachment during polymerase chain reaction (PCR) amplification flanked by 20–80 bases of random region [3]. The target of interest is first incubated with the library under specific ionic and temperature conditions. Library molecules that bind to the target are retained and amplified by PCR, while nonbinding library molecules are discarded. Negative or counter selections are often performed to increase the specificity of the library or direct the enrichment process away from binding to negative targets. Negative targets are often chosen for their structural similarities or the likelihood to coexist in the native environment with the target of interest. In this case, library molecules that bind to negative targets are discarded and those that do not bind are retained and amplified and thus completing one round of* in vitro* selection (Figure 1). It is expected that the library is enriched enough after approximately 12 rounds of SELEX. One or few nucleic acid MREs with high specificity and affinity toward their targets can be identified.

Both DNA and RNA MREs can conform into three dimensional structures, which include stem-loop, bulges, and/or hairpin regions and give rise to binding pockets for their respective targets [4]. There are reports suggesting that RNA MREs generally have a higher affinity for their target than their DNA counterparts [5]. However, unmodified RNA molecules are more susceptible to nuclease degradations than DNA. Modification on the 2′ hydroxyl of RNA molecules can increase their stabilities but may have negative impact on their binding affinities [6, 7]. It is also more difficult to amplify RNA MREs during selection, as reverse transcription to DNA must be performed prior to PCR. For these given reasons, there is a bigger hurdle to successfully identify and apply RNA MREs in molecular detection, and thus this review has chosen to focus on the discussion of ssDNA MREs in biosening applications.

Single-stranded DNA MREs have high affinity and specificity toward their targets that is comparable to antibodies. In addition, ssDNA MREs have several advantages over antibodies. Firstly, ssDNA MREs are more thermostable and can be reversibly denatured. This reusability is particularly desired for molecular sensing applications. Secondly, ssDNA MREs can be identified for targets that are nonimmunogenic or toxic to cells, as the SELEX process can be performed completely* in vitro* and independent of living systems. Lastly, identified ssDNA MREs with known sequences can be chemically synthesized at low cost and without batch to batch variations [8]. Different modifications such as thiol or amino functional groups can also be easily incorporated onto the 3′ and/or 5′ ends of oligonucleotides during synthesis and utilized for immobilization on solid platforms. Similarly, labeling molecules such as biotin or FITC can also be covalently attached and serve as reporters in sensing applications. The attractive features of ssDNA MREs allow researchers to investigate the translational application of biosensors. This review focuses on the recent advancements in the identification and biosensing application of ssDNA MREs specific for bacteria, viruses, their associated biomolecules, virulence factors, and selected biological and chemical toxins. Detection of these targets has been shown to be important in medical diagnosis, food safety, and environmental monitoring. Additionally, major principles in MRE based biosensors are briefly discussed.

## 2. *In Vitro* Selection of ssDNA Molecular Recognition Elements

### 2.1. General Methodology of SELEX

The general process of* in vitro* selection of ssDNA MREs starts from design and chemical synthesis of ssDNA library. ssDNA library consists of two predetermined constant regions for primer attachment during PCR amplification flanking a random region. This random region gives raise to the diversity of the library, which can be designated by 4^*n*^, where *n* is the number of bases in the random region. Longer random regions not only may result in increased library diversity, but also may risk inhibition of PCR amplification due to secondary structure formation. Therefore, the overall libraries lengths are usually designed to be less than 150 bases in total length, including a random region of 20 to 80 bases, and are chemically synthesized using phosphoramidite chemistry [3].

The SELEX process begins by incubating up to 10^15^ different ssDNA molecules with the target of interest. One of the key steps in the SELEX process is the separation of bound MREs from unbound MREs. The separation process is often achieved by target immobilization. Immobilization options include nitrocellulose membranes that can be used to adsorb protein targets [9] and histidine tags on recombinant proteins that can be with a metal affinity chromatography column [10]. However, ssDNA molecules may nonspecifically adsorb to immobilizing substrates. A round of negative selection is typically performed prior to the start of the first round of positive selection to reduce the nonspecific adsorption between the library and immobilizing substrates. Magnetic beads have also been used to immobilize a wide range of targets [11–14]. The terminal primary amine or a surface lysine on a protein can be used to conjugate onto carboxylic acid coated magnetic beads via EDC/NHS reactions. Small molecule targets or target analogs with available functional groups can also be biotinylated and immobilized on streptavidin coated magnetic beads based on the strong affinity between biotin and streptavidin [14, 15]. Magnets can then be used for the separation of bound and unbound molecules. However, this technique runs the risk of selecting MREs bound to magnetic beads and/or streptavidin. Sooter and coworkers successfully showed that competitive elution with free target can effectively isolate ssDNA MREs specific for the target of interest and not for the immobilizing substrates or analog molecules [14–16].

Amplification of the ssDNA library is also crucial to the success of the* in vitro* selection process. PCR conditions have to be determined and optimized before the selection process. After the retrieval of target bound ssDNA molecules for each round of selection, a small-scale PCR can be carried out to determine the cycles of PCR needed to successfully amplify the library. Large-scale PCR can subsequently be performed based on the determined number of reaction, and thus decreasing the chance of overamplification and the generation of undesired PCR amplicons.

It is necessary to obtain ssDNA from double-stranded PCR product prior to the subsequent rounds of selection. Several techniques have been shown to effectively isolate the single-stranded binding element from double-stranded DNA, such as asymmetric PCR, biotin-streptavidin separation, lambda exonuclease digestions, and size separation on denaturing urea polyacrylamide gel electrophoresis. Asymmetric PCR uses a different ratio of forward and reverse primer in the reaction mixture to generate both dsDNA and ssDNA allowing the two types of DNA molecules to be visualized and separated using agarose gel electrophoresis. The ssDNA is then excised and purified [17]. Biotin-streptavidin separation uses a biotin-tagged primer in the PCR amplification process to generate biotinylated dsDNA. The dsDNA can then be captured by streptavidin coated beads. The unbound strand of DNA can be retrieved using sodium hydroxide [18]. Lambda exonuclease can selectively digest a phosphorylated strand of the dsDNA in 5′ to 3′ direction. PCR reactions carried out with a phosphorylated reverse primer can be selectively digested by lambda exonuclease, leaving only the forward strand [19]. Modified primers can be used to create size differences between the forward and reverse strands and be detected by using urea denaturing PAGE, and subsequently ssDNA can be excised and purified [20].

### 2.2. Examples of Modified SELEX

The general process of SELEX has been modified over the past two decades. These modifications mostly focus on increasing the efficiency in separating bound and unbound MREs, increasing specificity of the selected MREs, eliminating the need for immobilizing target molecules, selecting against live whole cells, and decreasing the overall labor intensiveness of the SELEX process. Selected modified SELEX methods pertinent to this review are briefly discussed.

Negative or counter selection is incorporated into the normal SELEX process by introducing negative targets that have structural similarity to the target of interest or are likely to coexist in the target's environment. This modification is to increase the overall specificity of the library during selection and thus identify MREs that are highly specific to the target. Williams and coworkers identified ssDNA MRE target for herbicide, atrazine, with 2.1-fold higher binding affinity to atrazine than to a closely related herbicide, simazine, by introducing multiple negative selection rounds and increasing stringency during the selection [14]. This stringent negative selection scheme was utilized to obtain two other ssDNA MREs that bind to their respective targets with high affinity and specificity [15, 16].

Capillary electrophoresis can separate molecules based upon their charges. Target bound and unbound DNA molecules migrate at different rates due to differences in their overall charges, and therefore different species can be separated and collected at different time points. Mendonsa and Bowser were the first to use capillary electrophoresis to identify a ssDNA MRE specific for human IgE. Due to its high efficiency in separating different molecules, MREs can generally be identified in 4 to 6 rounds of capillary electrophoresis based SELEX (CE-SELEX) [21]. CE-SELEX can also select MREs bound to free targets in solution and without the need of immobilization. A variant of CE-SELEX utilizes nonequilibrium capillary electrophoresis of equilibrium mixtures (NECEEM) to achieve separation (Non-SELEX) has also been developed. In Non-SELEX, repetitive rounds of selection are performed without PCR amplification. Berezovski and coworkers were the first to use Non-SELEX to identify a high affinity MRE (*K*_*d*_: 0.3 nM) specific for hRas protein [22].

Park and coworkers developed an immobilization-free SELEX method based on *π*-*π* stacking interaction between DNA and graphene oxide (GO-SELEX). In GO-SELEX, ssDNA library is adsorbed on graphene oxide and then incubated with the target. In the presence of the target, a portion of the ssDNA library is released from graphene oxide and bind preferentially to the target, while unbound ssDNA remains adsorbed and can be separated by centrifugation [23]. This method was used to isolate ssDNA MREs specific for bovine viral diarrhea virus type 1 [24]. A high-throughput modification of GO-SELEX was also developed by Nguyen and coworkers to identify flexible ssDNA MREs that are specific for multiple pesticides with affinities in the nanomolar range [25]. Nutiu and Li developed a different target immobilization-free SELEX method using a ssDNA library containing a 15-base constant region, sandwiched by two random regions, and finally encompassed by two constant primer hybridization regions at both 3′ and 5′ ends [26]. The 15 bases constant region can hybridize with biotinylated complementary strand and can be captured by streptavidin coated beads. Binding of the ssDNA library to target molecules induces conformational changes, thus releasing the binding-strand from the complementary strand. This method has been adapted to screen for ssDNA MREs specific for multiple pesticides [27, 28].

FluMag-SELEX was developed by Stoltenburg and coworkers by immobilizing targets on magnetic beads, using fluorescently labeled forward primer during PCR amplification [29]. Magnetic separation of bound and unbound MREs is performed similarly to traditional magnetic bead based SELEX. However, the overall binding capacity of the library can be monitored precisely with the presence of fluorescence tag. The selection process can then be terminated when the overall library binding affinity toward the target reaches a plateau. A similar technique has been incorporated in single microbead SELEX described by Tok and Fischer. In their work, only 2 cycles of SELEX were performed to identify multiple ssDNA MREs specific for botulinum neurotoxin with low micro- to nanomolar *K*_*d*_ values [30]. The usage of fluorescence tag in the library is further investigated by Lauridsen and coworkers by performing a one-step selection against alpha-bungarotoxin [31].

Microfluidic chips are also being investigated to facilitate the SELEX process (M-SELEX). Microfluidic chips are capable of manipulating a very small amount of immobilized target on magnetic beads, thus achieving a more efficient separation of bound MREs [32]. Qian and coworkers were able to identify ssDNA MREs specific for Botulinum neurotoxin type A with low nanomolar binding affinity after only one round of selection [32, 33]. Recently, MREs with nanomolar binding affinity specific for whole influenza A/H1N1 virus were selected using M-SELEX [34].

Complex targets such as live mammalian and bacteria whole cells have become popular targets for selection. These types of selection are called cell-SELEX or whole cell-SELEX. Early works mostly focused on identifying MREs specific for tumor cells [35–38]. The general methodology of cell-SELEX is very similar to traditional SELEX, but fluorescence-activated cell sorting (FACS) can be utilized to achieve a very high level of separation of MRE bound and unbound cell targets. Multiple pathogenic bacteria genera, such as* Salmonella*,* Pseudomonas*,* Staphylococcus*,* Listeria*, and* Escherichia* have been chosen as a selection target. The selection and biosening application of ssDNA MREs targeting bacteria, viruses, and associated biomolecules are discussed in the following section.

### 2.3. Single-Stranded DNA MREs Targeting Bacteria

Single-stranded DNA MREs targeting bacteria can be classified into two general categories, (1) targeting whole cells with known or unknown molecular targets and (2) targeting predefined bacteria cell surface targets or bacteria spores (Table 1).

Multiple virulent strains of the gram-negative bacteria,* Escherichia coli*, have been chosen as targets for the selection of specific ssDNA MREs due to their enterotoxigenic effects and the potential of contaminating food and water [39]. Peng et al. enriched ssDNA MRE library specific for* E. coli* K88 whole bacteria [40]. They also developed a sandwich detection system, in which biotinylated antibodies targeting the K88 strain were immobilized on magnetic beads as the capturing element and the 5′ FITC labeled ssDNA library from round 13 selection served as the reporter in a fluorescent assay. A lower limit of detection (LOD) of 1100 CFU/mL was achieved in pure culture. Artificial contaminated fecal samples were also tested with a LOD of 2200 CFU per gram. However, no individual ssDNA MRE was able to achieve the same degree of binding affinity as the whole library and ssDNA MRE with high affinity and specificity against K88 fimbriae protein was selected after 11 rounds [41]. A fluorescence binding assay was used to obtain the affinity of the selected MRE candidates. The reported equilibrium dissociation constant (*K*_*d*_) for the best candidate MRE was 25 ± 4 nM. Kim et al. performed 10 rounds of selection against a fecal strain of* E. coli *along with multiple negative selections against other species of bacteria. They identified four candidate sequences with high affinity for the target strain. All four candidates were highly selective against negative target bacteria. However, they all showed cross-binding activity with other strains of* E. coli*. This suggested that the selected candidates potentially bound to common antigens expressed in multiple strains of* E. coli *[42]. Savory et al. identified ssDNA MRE with high specificity and affinity (*K*_*d*_ = 110 nM) for an uropathogenic strain of* E. coli*. Quantitative PCR was used to monitor the SELEX process in order to minimize the number of rounds of SELEX required. After 5 rounds of SELEX, a selected ssDNA MRE containing a guanine-quadruplex sequence motif showed low cross-binding activities toward other strains of* E. coli* [43]. In addition to selecting whole* E. coli* bacteria as targets, outer membrane protein from* E. coli* 8739 (Crook's strain) and lipopolysaccharide from O111:B4 strains were also chosen as targets for selection. A fluorescence resonance energy transfer (FRET) assay was developed to detect* E. coli* 8379 with a LOD of 30 CFU/mL [44]. The ssDNA MRE targeting lipopolysaccharide showed antibacterial effects on both O111:B4 and K12 strains [45]. However, *K*_*d*_ values were not reported in either study.

Several ssDNA MREs have been selected against species of foodborne bacteria including* Salmonella*,* Listeria*, and* Vibrio*. Dwivedi et al. identified ssDNA MRE specific for whole cell* Salmonella enterica *serovar Typhimurium with a reported *K*_*d*_ of 1.73 ± 0.54 *μ*M after eight rounds of selection [46]. Two rounds of negative selection against a mixture of nontarget bacteria were also performed to enhance the selectivity of the library. A detection application was developed using immobilized biotinylated MREs on streptavidin coated magnetic beads as the capturing elements and was coupled with quantitative PCR. The reported LOD of this assay was between 100 to 1000 CFU in a 290 *μ*L sample volume. Duan et al. performed a similar selection on the same organism with nine rounds of target selection and two rounds of negative selection against mixtures of nontarget bacteria [47]. The best candidate ssDNA MRE had a *K*_*d*_ value of 6.33 ± 0.58 nM and high specificity based upon flow cytometry analysis. A fluorescence bioassay achieved a LOD of 25 CFU/mL. Another similar study performed by Moon et al. showed relatively high affinities and specificities of selected candidate sequences after ten rounds of target and six rounds of negative selections. However, no *K*_*d*_ values were reported in the study [48]. Outer membrane proteins of* Salmonella enterica *serovar Typhimurium were chosen as selection target by Joshi et al. In that study seven rounds of selection were performed with three rounds of negative selection against* E. coli* outer membrane proteins and lipopolysaccharides. A magnetic bead based quantitative real-time PCR assay was developed using immobilized ssDNA MRE as the capturing element. Food and environmental samples were tested to demonstrate the translational usage of the assay. A LOD of less than 10 CFU per gram of artificially contaminated bovine feces was reported. Additionally, 10 to 100 of CFU were detected in 9 mL of artificially contaminated whole carcass chicken rinse sample solution in a pull-down assay [49]. Two recent studies identified ssDNA MREs specific for two serovars of* Salmonella*, Typhimurium, and Enteritidis [50, 51]. Park et al. truncated out the random region (29 to 30 mer) of selected candidates and identified three ssDNA MREs with *K*_*d*_ values in micromolar range toward their respective serovars after ten rounds of mixed target and counter target selection. Poly-D-lysine was conjugated to the selected MREs and achieved an approximately 20- to 100-fold enhancement in their binding affinities [51]. Kolovskaya et al. also performed a similar selection on the two serovars of* Salmonella* [50]. After twelve rounds of selection, two ssDNA MREs with *K*_*d*_ values range in nanomolar were identified (Enteritidis: *K*_*d*_ = 7 nM; Typhimurium: *K*_*d*_ = 25 nM). Both selected MREs were able to demonstrate a bacteriostatic effect in their respective bacterial cultures. An antibiotic-resistant serovar of* Salmonella enterica*, Paratyphi A, was chosen as target by Yang et al. A total of thirteen positive rounds and four negative rounds of selection were performed to identify an MRE with high affinity (*K*_*d*_ = 47 ± 3 nM) and specificity toward Paratyphi A. LOD of 1000 CFU/mL was achieved using chemiluminescence assay based on self-assembled single-walled carbon nanotubes and DNAzymes-labeled MRE as detection elements [52]. MRE with high specificity toward* Salmonella* O8 was identified by Liu et al. after eleven rounds of positive and two rounds of negative selection. The selected MRE had a reported *K*_*d*_ of 32.04 nM. A preliminary fluorescent* in situ* labeling assay was developed with the MRE. However, no LOD was reported [53].

Consumption of uncooked or undercooked seafood contaminated by* Vibrio *bacteria can lead to food poisoning [54]. Two different species,* Vibrio parahaemolyticus *and* Vibrio alginolyticus* were chosen as selection targets. Nine rounds of cell-SELEX using flow cytometry were carried out to identify ssDNA MRE with high affinity and specificity for* Vibrio parahaemolyticus* (*K*_*d*_ = 16.88 ± 1.92 nM) [55, 56]. Tang et al. performed 15 rounds of cell-SELEX on inactivated* Vibrio alginolyticus*. Negative selection was performed every third positive target round to improve the library specificity. The study did not characterize affinities and specificities of candidate ssDNA MREs from the last round of selection. Instead, the whole library was characterized to have a *K*_*d*_ value of 27.5 ± 9.2 nM and was highly specific toward the target. The enriched library was able to detect 100 CFU/mL of the bacteria based on a PCR amplification assay [56].

*Listeria monocytogenes *is a foodborne gram-positive bacterium that can cause serious illnesses and even death. FDA and European Union both have zero tolerance of* L. monocytogenes* in ready-to-eat food products. Suh et al. conducted two studies to identify ssDNA MREs specific for* L. monocytogenes *[57, 58]. In their earlier study, MRE with a micromolar *K*_*d*_ value was identified after six rounds of positive and two rounds of negative selections. The MRE showed low cross-binding to negative target bacteria but had similar binding affinity for other members of the* Listeria* genus. A magnetic bead based capture assay coupled with quantitative PCR was developed. The assay was able to detect less than 60 CFU in 500 *μ*L of binding buffer containing a mixture of non-*Listeria* bacteria [58]. In their later study, the affinities of selected candidate MREs were improved with reported values of *K*_*d*_ in the nanomolar range and were specific for the target bacteria at different growth phases [57]. Duan et al. performed similar whole cell* in vitro* selection on* L. monocytogenes. *The selected MRE had high affinity (*K*_*d*_ = 48.74 ± 3.11 nM) and was highly specific toward the target. A fluorescent cross-binding assay showed significantly lower binding activities toward negative bacteria targets and other bacteria species in the* Listeria* genus. A sandwich fluorescent bioassay was developed and demonstrated a LOD of 75 CFU/mL [59]. Most recently, Liu et al. performed eight rounds of selection to identify ssDNA MREs specific for* L. monocytogenes*. The best candidate MRE reported to have a *K*_*d*_ value of 60.01 nM and had high specificity. A fluorescent based detection assay was developed to enable the observation of binding between the MRE and target bacteria using fluorescent microscope, but the LOD was not reported [60].

Ohk et al. selected ssDNA MRE specific for internalin A of* L. monocytogenes*. Internalin A is a major invasion protein expressed on the cell surface of* L. monocytogenes* [61]. A highly specific sandwich style fiber optic biosensor was developed by using the selected MRE and antibody. A reported LOD of 1000 CFU/mL was achieved. The sensor also successfully detected the bacteria in artificially contaminated ready-to-eat meat products. However, affinity data was not reported in the study [62].

*Shigella dysenteriae* is a gram-negative bacterium that causes severe epidemic diarrhea in many countries [63]. Duan et al. used cell-SELEX methodology to identify ssDNA MRE specific for* S. dysenteriae* [47, 55, 59, 64]. The best candidate MRE had a reported *K*_*d*_ value of 23.47 ± 2.48 nM and low cross-binding activities toward negative bacteria targets. A fluorescent based detection assay demonstrated a LOD of 50 CFU/mL [64].

*Campylobacter jejuni *is a highly infectious gram-negative bacterium that is one of the leading causes of acute diarrheal sickness worldwide [65]. Bruno et al. performed an* in vitro* selection by extracting surface proteins of* C. jejuni* and immobilizing them on magnetic beads. No values of *K*_*d*_ were reported in the study. However, a fluorescent assay based on magnetic beads and quantum dot was developed to detect the bacteria in different food matrices. The assay showed low cross-binding activities with other species of bacteria but was not able to distinguish between bacteria in the* Campylobacter *genus. The reported LODs were 2.5 CFU and 10 to 250 CFU in buffer solution and in different food matrices, respectively [66]. CE-SELEX was employed by Stratis-Cullum et al. to identify MREs specific for* C. jejuni*. Killed bacteria were used as target in their study. A qualitative capillary electrophoresis immunoassay was developed with a LOD of 6.3 × 10^6^ cells/mL [67]. Dwivedi et al. performed cell-SELEX on live* C. jejuni*. A total of ten positive rounds and two negative rounds were carried out to identify ssDNA MREs with high affinity and specificity toward the target bacteria (*K*_*d*_ = 292.8 ± 53.1 nM) [68].

Bacteria that are associated with common infectious diseases, such as* Streptococcus*,* Staphylococcus*, and* Pseudomonas*, are also popular targets for* in vitro* selection. Identification of MREs targeting infectious bacteria could be potentially used to facilitate diagnosis and thus decreasing the time between culture collections to specific antibiotic treatment.

Savory et al. performed cell-SELEX on* Proteus mirabilis*, a common cause of catheter associated urinary tract infections in long-term catheterized patients. MREs specific for two different strains of* P. mirabilis* with low nanomolar range *K*_*d*_ values were identified after 6 rounds of* in vitro* selection. Additionally, an* in silico* maturation (ISM) process was performed to increase the specificity of the selected MRE. It was reported that a 36% higher specificity was achieved after the ISM process [69]. This same technique was again employed to select MRE specific for* Streptococcus mutants*, the main causative pathogen of dental caries. The affinity of the identified MRE was improved up to 16-fold and the specificity was increased 12-fold after ISM. A gold colloids based colorimetric flow-through assay was developed and demonstrated the detection* S. mutants* in the range of 10^5^–10^8^ CFU/mL [70].

*Streptococcus pyogenes* (Group A* Streptococcus*) is often the causative pathogen of a wide range of infectious diseases, such as streptococcal pharyngitis and streptococcal toxic shock syndromes [71]. Different M-types of live* S. pyogenes* were chosen for selection by Hamula et al. After 20 rounds of target selection, the two best candidate MREs yielded high affinity for Group A* Streptococcus* (*K*_*d*_ = 9-10 nM). It was noteworthy that the candidate MREs showed good specificities, even though the authors did not perform any negative selections [72].

*Staphylococcus aureus *is a gram-positive bacteria associated with numerous of infections in human [73]. Cao et al. selected a panel of ssDNA MREs specific for* S. aureus *after several rounds of target and counter target selection. The reported *K*_*d*_ values of individual candidate MREs were in the nanomolar range with high specificity. The study showed that the combination of the panel of MREs yielded a better sensitivity in recognizing* S. aureus* than any single MRE [74]. Change et al. selected two ssDNA MREs with high affinities and specific toward* S. aureus* (*K*_*d*_ = 35 and 129 nM). The reported values of *K*_*d*_ improved to 3.03 and 9.9 nM, respectively, after thiol-modification and conjugation to gold nanoparticles. Subsequently, the MRE conjugated gold nanoparticles were utilized to capture target bacteria and a resonance light-scattering signal demonstrated the detection of single* S. aureus* cell in 1.5 hours [75].

*Pseudomonas aeruginosa* is a gram-negative bacterium that is commonly associated with nosocomial infections [76, 77]. Wang et al. performed 15 rounds of positive and 2 rounds of counter target selection to identify ssDNA MREs with *K*_*d*_ values in the low nanomolar range. The selected MRE showed negligible binding to counter bacteria cell targets. A fluorescence* in situ* hybridization (FISH) assay was developed to show rapid detection of* P. aeruginosa*. However, the detection ranges were not reported [78].

*Mycobacterium tuberculosis* is the etiologic pathogen for tuberculosis [79]. Chen et al. reported ssDNA MRE with an apparent association constant (*K*_*a*_) between 10^5^-10^6^ M and was highly specific. The authors reported an antibacterial effect of the selected MRE with both* in vitro* and* in vivo* models [80].

Highly infectious bacteria and bacteria spores have been considered as potential biological warfare agents, and it is important to detect these biological threats rapidly [81]. Bruno and Kiel 1999 performed an* in vitro* selection of ssDNA MREs targeting* Bacillus anthracis* spores, the causative agent of anthrax. Autoclaved anthrax spores were used in the selection. MRE-magnetic bead electrochemiluminescence sandwich assay was developed with a reported detection range of 10–10^6^ spores [82]. Ikanovic et al. performed a selection of ssDNA MREs specific for* Bacillus thuringiensis* spores, a closely related species to* B. anthracis*. In this study, the methodology was adopted from Bruno and Kiel 1999. A fluorescent assay based on cadmium selenide quantum dots was developed with a reported detection limit at about 1000 CFU/mL [83]. Bruno and Carrillo 2012 revisited the selection of* Bacillus* spores. In this later study, anthrose sugar on anthrax spores was chosen as target for selection. MRE beacon based on fluorescent signals was developed and generated strong signal at spore concentrations greater than 30,000 spores/mL. The authors also compared the MRE sequences pattern to previous studies and identified similarities in sequences composed of T/G rich bases. It was also reported that MREs specific for whole spores did not generate fluorescent signals in the presence of anthrose sugar, suggesting that the selected spore specific MREs possibly bound to a different epitope and warranting further research [84].

*Francisella tularensis* is an encapsulated, gram-negative coccobacillus that is highly infectious. Reports show as few as 25 organisms in aerosol can cause diseases [85]. Vivekananda and Kiel performed ten rounds of selection on* Francisella tularensis *subspecies* japonica* bacterial antigen. A cocktail of 25 ssDNA MREs was reported to have high specificity toward the target bacteria. MRE modified enzyme linked immunosorbent assay was developed, and demonstrated binding to the target and other subspecies of* F. tularensis *but not to other species of bacteria and chicken lysozyme or chicken albumin. In addition, the assay was able to achieve better sensitivity then traditional ELISA using anti-tularemia antiserum and anti-tularemia polycolonal antibodies. The reported LOD is 1700 bacteria/mL [86].

Peptidoglycan is a macromolecule universally expressed on bacteria outer cell wall [87]. Ferreira et al. identified two ssDNA MREs with sub- to low micromolar *K*_*d*_ values that can bind specifically to both gram-positive and gram-negative bacteria. Neither MRE bounded to human fibroblasts or* Candida albicans* and could potentially be used as generic detection elements for bacteria [88].

Lipopolysaccharide (LPS or endotoxin) is expressed in the outer membrane of gram-negative bacteria and can illicit strong immune response upon entering into mammalian cells. [89, 90] Kim et al. used nonequilibrium capillary electrophoresis of equilibrium mixtures (NECEEM) based non-SELEX to identify multiple ssDNA MREs with high affinities toward lipopolysaccharide in only three rounds of selection. Selected MREs also demonstrated very low cross-binding activities to bovine serum albumin and other intracellular molecules, such as DNA, RNA, glucose, and sucrose, in an electrochemical assay. This assay resulted in a target detection range of 0.01 to 1 ng/mL [91].

### 2.4. Single-Stranded DNA MREs Targeting Viruses

There is a wealth of literature describing ssDNA MREs targeting various virus life cycle regulator proteins with the purpose of therapeutic application. In contrast, there is a lesser amount of research on ssDNA MREs for virus biosensing application (Table 2). For the focus of this review, those MREs with therapeutic applications are listed in the following table without further detail discussions (Table 3).

In recent years, there has been an increase in the interest in the application of ssDNA MREs for virus detection. Human noroviruses are the leading cause of acute gastroenteritis worldwide. Current detection methods for the virus are time-consuming and labor intensive [92]. Giamberardino et al. performed nine rounds of* in vitro* selection on murine norovirus. The best candidate MRE was reported to have affinity in the low picomolar range. It displayed cross-binding activity with human norovirus but not to a structurally similar virus,* feline calicivirus*. An electrochemical sensor using a gold nanoparticle modified screen-printed carbon electrode was developed with a reported LOD of 180 virus particles [93]. Escudero-Abarca et al. performed selection on multiple strains of human noroviruses, including Snow Mountain virus and Norwalk virus. Candidate MREs showed high binding affinities that were comparable to commercially available antibodies. The best MRE was also able to show binding to specific virus strains in human fecal samples. A reported LOD of 10 virus RNA copies was achieved in artificially contaminated lettuce by using immobilized biotinylated MRE on streptavidin magnetic beads coupled with real-time quantitative PCR [94]. The capsid protein VP1 of Norovirus genotype II.4 was chosen to be the target for selection by Beier et al. After twelve rounds of selection, surface plasmon resonance analysis was used to show the high specificity of the selected MRE. Computer simulation was used to characterize the binding interaction between VP1 and candidate MREs. However, the authors did not report the *K*_*d*_ in the study [95].

Influenza virus is the causative agent for many upper respiratory diseases and can potentially cause pandemics with high mobility and mortality [96]. Lai et al. used M-SELEX to identify ssDNA MRE target influenza A H1N1 with high affinity (*K*_*d*_ = 55.14 ± 22.40 nM). A magnetic bead based florescent assay achieved a reported LOD of 6.4 × 10^−3^ HAU. When the bead capturing method was coupled with RT-PCR, the fluorescent signal remained detectable in virus spiked clinically relevant matrices, including throat swab samples, sputum samples, and serum samples [34]. Wang et al. performed an* in vitro* selection specific for influenza virus H5N1. Purified hemagglutinin (HA) protein was used as target for the first four rounds and then inactivated whole H5N1 viruses were used from round five to thirteen. The best candidate MRE displayed high affinity (*K*_*d*_ = 4.65 nM) with only minimal cross-binding activities on other avian influenza virus strains. A dot blot assay was developed with a LOD of 1.28 HAU, which was comparable to anti-H5 antibody. The dot blot assay also demonstrated the detection of the target virus in spiked chicken and duck swab samples [97]. Shiratori et al. chose recombinant influenza A HA protein as a target for selection. After ten rounds of target selection, candidate sequences showed high binding affinities with reported *K*_*d*_ values in the low nanomolar range. A sandwich enzyme linked aptamer assay (ELAA) was developed and showed similar binding responses on three strains of influenza A, H5N1, H1N1, and H3N2. However, the LOD was not determined [98].

A novel coronavirus caused a severe acute respiratory syndrome (SARS) outbreak in 2002 to 2003 [99]. Cho et al. identified ssDNA MREs specific for the SARS coronavirus nucleocapsid protein. After twelve rounds of positive selection, the best candidate MRE had a reported *K*_*d*_ of 4.93 ± 0.3 nM. MRE modified Western blot showed a comparable detection level to nucleocapsid antibody based assay. However, the authors did not show cross-reactivity of the selected MREs toward other viral proteins [10].

GO-SELEX was utilized to identify ssDNA MREs specific for bovine viral diarrhea virus. After five rounds of positive and negative selections, three best candidate MREs had reported *K*_*d*_ values of 4.08 × 10^4^, 4.22 × 10^4^, and 5.2 × 10^4^ TCID_50_/mL, respectively, by SPR kinetics analysis. All candidate MREs showed very high specificity toward the target. A sandwich SPR detection assay was developed wherein a biotinylated MRE was immobilized on streptavidin coated gold chip as the capturing MRE, and a second different MRE with thiol modification was conjugated to gold nanoparticle as the reporting MRE. A LOD of 800 copies of virus/mL was reported with this assay [24].

Hepatitis C virus (HCV) envelope surface glycoprotein E2 was chosen as target for selection by Chen et al. E2 protein was expressed on a murine colon carcinoma cell line, CT26 cells, and used as a target for positive selection. The native CT26 cells were used as counter target. After 13 rounds of selection, the best candidate MRE showed high affinity and specificity toward E2-positive cells. An ELISA virus capture assay was developed by using biotinylated MRE as reporter and demonstrated the detection of HCV in clinical human serum samples. In addition, the MRE, termed ZE2 also displayed therapeutic effect by inhibiting E2 protein binding to CD81 and blocking HCV infection of human hepatocytes* in vitro* [100].

Dengue virus is a member of family* Flaviviridae*, genus* flavivirus*. It is a mosquito-borne RNA virus that can cause gangue fever, dengue hemorrhagic fever, and dengue shock syndrome [101]. Gandham et al. used recombinant dengue virus type-2 envelope protein domain III as target of interest to perform an* in vitro* selection of thiophosphate ester modified ssDNA MREs. After five rounds of target selection, the best MRE had a reported *K*_*d*_ of 154 ± 40 nM, but no cross-binding experiments were performed [102].

CE-SELEX was performed by Mosing et al. to identify ssDNA MREs specific for HIV reverse transcriptase. After only four rounds of selection, the best candidate MRE had an ultrahigh affinity with a reported *K*_*d*_ in the picomolar range. Interestingly, there were no identifiable consensus sequence families in the round four ssDNA library. The authors claimed that the selected MRE had the highest affinity for the target of interest when compared to MREs selected by other methods and suggested that multiple ultrahigh affinity MREs might exist in the enriched library [103].

### 2.5. Single-Stranded DNA MREs Targeting Toxins

#### 2.5.1. Biological Toxins/Virulence Factors

Secretory proteins, virulent factors, exotoxins, or small molecule toxins from bacteria, fungus, and other organisms are important biomarkers in medical diagnosis, environmental monitoring, and food safety surveillance. The following section discusses recent studies in the identification and biosening application of ssDNA MREs specific for biological toxins (Table 4).

*Staphylococcus aureus* can secrete a group of thermostable enterotoxins that have been shown to contaminate food. Reports suggest that these toxins are a common cause of foodborne illnesses [104]. There are many types and subtypes of staphylococcus enterotoxins. Bruno and Kiel first selected ssDNA MREs that bind to enterotoxin B by using magnetic bead immobilized target. An electrochemiluminescence assay was developed to demonstrate a detection limit of less than 10 pg of enterotoxin B. However, no kinetic data or crossing-binding profiles were presented in the study [105]. DeGrasse recently identified ssDNA MRE specific for enterotoxin B after fourteen rounds of mixed target and negative targets selection. MRE based precipitation assay was used to analyze the selectivity of candidate MREs in cell-free culture supernatant from multiple strains of* S. aureus*. The high selectivity of candidate MREs was confirmed by capturing only the target toxin in the precipitation assay. However, no quantitative binding data was presented in the study [11]. Enterotoxin subtype C1 was chosen as a target for selection by Huang et al. After twelve rounds of selection, the best candidate MRE demonstrated high affinity for enterotoxin C1 (*K*_*d*_ = 65.14 ± 11.64 nM). Cross-binding experiments showed that the selected MRE had high specificity and low cross-binding activities on staphylococcus enterotoxin A, enterotoxin B, and other protein molecules. A graphene oxide based fluorescence detection assay was developed and achieved a reported LOD of 6 ng/mL in artificially contaminated buffer-diluted milk samples [106].

Bruno and Kiel 2002 selected ssDNA MRE against cholera toxin. An enzyme linked colorimetric assay showed a detection limit of less than 10 ng of cholera toxin and electrochemiluminescence assay shows a detection limit of less than 40 ng. However, affinity, crossing-binding data, and MRE sequences were not presented in the study [105].

Toxigenic strains of* Clostridium difficile *can produce toxins A and toxin B, which are the contributing factor of* C. difficile* induced diarrhea. Rapid diagnosis of the condition is crucial in facilitating patient recovery and disease control [107]. Some strains of* C. difficile* also secret a binary toxin that can inhibit actin polymerization [108]. Ochsner et al. selected several slow off-rate modified ssDNA MREs (SOMAmer) specific for toxins A, B, and binary toxin. Several DNA libraries with modifications, such as 5-benzylaminocarbonyl-dU (BndU), 5-naphthylmethylaminocarbonyl-dU (NapdU), 5-tryptaminocarbonyl-dU (TrpdU), 5-phenylethyl-1-aminocarbonyl (PEdU), 5-tyrosylaminocarbonyl-dU (TyrdU), or 5-(2-naphthylmethyl) aminocarbonyl (2NapdU) were used in selections. Truncated recombinant toxins were used as targets. Equilibrium dissociation constants of selected SOMAmers were in pico to nanomolar range. The affinities for native toxins were slightly lower but were remain in the low nanomolar range for majority of the candidate sequences. Pull-down capture, dot blots, and antibody sandwich assays were developed with a reported LOD of 300 pg/mL. Selected SOMAmers were able to detect all three toxins in cell-free culture supernatants of toxigenic* C. difficile *[109]. Ochsner et al. performed another* in vitro* selection on* C. difficile* binary toxin with sandwich SELEX. The advantage of sandwich SELEX is to select SOMAmer pairs that target different epitopes of the target protein. The reported *K*_*d*_ values of selected SOMAmers ranged from 0.02 to 2.8 nM. A SOMAmer sandwich assay was developed with a reported LOD in the low picomolar range. The authors claimed that these studies showed the high potential for the development of sensitive and specific diagnostic kits [110].

Hong et al. performed twelve rounds of positive* in vitro* selection against* C. difficile* toxin B and eleven rounds of negative selection. SPR binding study determined the selected ssDNA MRE had a *K*_*d*_ value of 47.3 ± 13.7 nM. Fluorescence plate based cross-binding assay showed the selection ssDNA MRE was two to five times more selective on toxin B than negative targets. A proof-of-concept modified ELISA using ssDNA as the toxin capturing element was developed and able to detect toxin B at 50 nM concentration in human fecal matter [111].

Tuberculosis (TB) remains to be a challenging disease in developing countries. Recent discovery of multidrug resistant strains of* Mycobacterium tuberculosis* has further increased public concerns, however, current diagnostic techniques for TB are either time-consuming or insensitive [112–114]. Rotherham et al. performed a selection on CFP-10.ESAT6 heterodimer, a specific biomarker for TB infections. After six rounds of selection, SPR binding studies showed that candidate ssDNA MREs had affinities in the nanomolar range. One of the candidate MRE was tested in an enzyme linked oligonucleotide assay (ELONA). The authors reported that the assay had 100% sensitivity and 68.75% specificity in clinical sputum samples using Youden's index. However, the time needed for assay completion and crossing-binding activities to other antigens were major limitations of the assay [9]. Tang et al. performed a selection on the same CFP-10.ESAT6 heterodimer. After seventeen rounds of selection, *K*_*d*_ values of candidate MREs were in the low nanomolar range. Two ssDNA MREs (CE24, CE15) were used in an ELONA assay. The reported sensitivity and specificity of CE24 MRE based ELONA were 100% and 94.1%, respectively. CE15 MRE based ELONA had a lower sensitivity of 89.6%, but the specificity was the same. Assays were tested both pulmonary and extrapulmonary with serum samples from TB patients [115].

MPT64 is a secreted protein of* M. tuberculosis* and can be used as biomarker for active TB infections [116]. Qin et al. performed twelve rounds of selection on MPT64. Affinities of truncated candidate ssDNA MREs, containing only a 35-base central random region, were qualitatively observed using streptavidin-horse radish peroxidase (HRP) binding to protein-bound biotin-tagged MREs. A colorimetric sandwich assay using two different MREs was developed to detect the presence of MPT64 in culture filtrates. The sandwich assay achieved sensitivity and specificity of 86.3% and 88.5%, respectively [117].

Protective antigen (PA) is a secreted virulence factor of* Bacillus anthracis* that binds to anthrax toxin receptors on mammalian cells and subsequently causes cell dysfunction and death [118]. Cella et al. utilized CE-SELEX to identify ssDNA MRE targeting PA with high affinity and specificity. After six rounds of CE-SELEX, the best candidate had a reported *K*_*d*_ of 112 nM. An electrochemical biosensor was developed by immobilizing 5′ amino modified MRE on 1-pyrenebutanoic acid succinimidyl ester (PASE) modified single wall carbon nanotubes (SWNT). The sensor showed low cross-binding activity toward human and bovine serum albumin at 100 nM concentration. The sensor surface could be regenerated using 1 *μ*L of 6 M guanidine hydrochloride for 15 minutes followed by a wash with 10 mM phosphate buffer. A reported LOD of 1 nM was achieved [119]. Choi et al. performed an* in vitro* selection on PA. After eight rounds of selection, four candidate sequences had high affinities for PA (*K*_*d*_ in low nanomolar range), and two of the four candidates had low cross-binding activities toward bovine serum albumin and bovine serum [120].

Botulinum neurotoxins (BoNT) are produced by* Clostridium botulinum*. In addition to its medical uses, it can also cause serious foodborne illness and may potentially be used as a biological weapon [121]. Tok and Fischer used a novel single microbead SELEX to perform selection of ssDNA MREs specific to aldehyde-inactivated BoNT type A toxin (BoNT/A-toxoid) and BoNT type A heavy chain peptide (BoNT/A Hc-peptiod). Targets were immobilized onto Ni-NTA agarose or amine-functionalized polystyrene TentaGel beads. A single target-immobilized microbead was incubated with the ssDNA library and retrieved for PCR amplification of bound ssDNA molecules. After only two rounds of selection, five candidate sequences specific for BoNT/A Hc-peptiod had *K*_*d*_ values ranging from 1.09 *μ*M to 4.20 *μ*M. Three candidate sequences specific for BoNT/A-toxoid had *K*_*d*_ values ranging from 3 nM to 51 nM. The authors reported that all MREs specific to BoNT/A Hc- peptoid were able to competitively inhibit the binding between the toxin peptide and anti-BoNT antibody and potentially be used as a therapeutic agent [30].

Lou et al. utilized a novel microfluidic device to facilitate the partitioning of a small volume of target coated magnetic beads (M-SELEX). The library achieved a very high overall affinity (*K*_*d*_ = 33 ± 8 nM) against BoNT/A light chain after only one round of selection. Four candidate sequences had a range of *K*_*d*_ values between 34 and 86 nM. The authors claimed that their M-SELEX could be readily adapted to any bead immobilized targets or whole cell target [33]. Bruno et al. immobilized BoNT/A light chain on magnetic beads and performed 10 rounds of selection. The best candidate MRE was fluorescently tagged and used as a reporter for target detection. The reported LOD of 1 ng/mL was achieved in buffer. However, the MRE reporter also bound to structurally similar targets, BoNT type B, type E holotoxins, and heavy or light chain components, in a soil dilution. The author compared their MRE sequence to previous ssDNA MRE specific for BoNT and found consensus short sequence segments. This suggested that the binding between BoNTs and MREs may be conserved within these consensus segments [122].

Microcystin is a hepatotoxin produced by cyanobacteria. Three different analogs of microcystin were used in the study performed by Nakamura et al. Microrocystin LR, containing a leucine substituent, was immobilized and used for twelve rounds of target selection. However, surface plasmon resonance binding data indicated a higher binding level between the selected MRE and microcystin YR, an analog containing a tyrosine substituent. There was also significant binding to microcystin RR, an analog containing an arginine substituent. The reported binding affinity (*K*_*a*_) was low, at approximately 10^3^ M^−1^. This early work did not demonstrate the high affinity and specificity properties of MREs; however, it did show the possibility of using MREs as a binding molecule in a label-free detection system [123].

Cylindrospermopsin (CYN) is another water soluble and heat stable alkaloid secreted by a large group of fresh water cyanobacteria. It has a variety of toxic effects in human bodies upon exposure to cylindrospermopsin usually through drinking water or food [124]. Elshafey et al. recently selected ssDNA MRE with high affinity and specificity toward CYN, with a reported *K*_*d*_ of 88.78 nM. Circular dichroism measurements showed that MRE had a conformational change upon binding to CYN. This property was exploited in a label-free impedimetric biosensor. The reported LOD of the sensor was 100 pM with a linear range of 80 nM. It also showed negligible responses toward coexistent cyanobacterial toxins of microcystin-LR and Anatoxin-a. CYN was recoverable in a spike test with tap water [125].

Saxitoxin is a small neurotoxin produced by few dinoflagellates and certain cyanobacteria that affect marine organisms [248]. Handy et al. were the first to select ssDNA MRE against target saxitoxin. In their study, saxitoxin was conjugated to keyhole limpet hemocyanin (KLH) via a spacer compound, 2,2′-(ethylenedioxy)bis(ethylamine), or JEFFAMINE and then the protein-toxin conjugate immobilized on magnetic beads. Ten rounds of selection were performed, and negative selection against KLH-bead was carried out from round four to the round ten, in order to decrease nonspecific binding to KLH and beads. One candidate sequence was analyzed by SPR and demonstrated a concentration-dependent and selective binding to saxitoxins. However, the *K*_*d*_ of the selected MRE was not presented in the study [145].

Okadaic acid (OA) is a phycotoxin produced by Dinophysis and Prorocentrum algae. It can accumulate in shellfish due to its lipophilic and heat-stable nature. Human consumption of OA can lead to a variety of gastrointestinal symptoms [249]. Eissa et al. identified ssDNA MRE with high affinity and specificity toward OA after eighteen rounds of mixed target and negative target selection. The candidate MRE with the highest affinity (*K*_*d*_ = 77 nM) was chosen for circular dichroism analysis. A conformational change in the MRE was observed upon binding of OA. A label-free electrochemical impedimetric biosensor was developed with this MRE and achieved a LOD of 70 pg/mL. It demonstrated no cross-binding activity toward structurally similar toxins, including dinophysis toxins-1 and -2 and microcystin-LR [12].

Ochratoxin A (OTA) is a mycotoxin produced by members of the* Aspergillus* and* Penicillium* genera. It is a nephrotoxin and has potential carcinogenic effects in humans. It has been shown as a contaminant in many food products, such as grains and wine [250]. However, the current detection method for OTA is both expensive and time-consuming [251]. Cruz-Aguado and Penner identified ssDNA MRE specific for OTA after thirteen rounds of selection. The best candidate MRE reported had a *K*_*d*_ value of 200 nM. It did not bind nonspecifically to warfarin, N-acetyl-L-phenylalanine, or ochratoxin B in a fluorescent based cross-binding assay [146]. Subsequently, the authors developed a detection system based on a fluorescence polarization displacement assay. The author reported that the assay was sensitive to OTA but not to warfarin and N-acetyl-L-phenylalanine, with a LOD of 5 nM. However, the detection assay did not test ochratoxin B (OTB) binding activity or sensitivity in food sample [147]. Barthelmebs et al. also selected ssDNA MRE specific for OTA. Several candidate MREs were identified after fourteen rounds of selection. After binding and cross-binding analysis, the best candidate had a *K*_*d*_ value of 96 nM with minimal binding to OTB and phenylalanine. It was incorporated into an ELISA and ELAA assays for the detection of OTA spiked in pretreated wine samples. Different ELAA and ELISA assays were compared, and a direct competitive ELAA had the lowest detection limit of 1 ng/mL with the shortest analysis time of 125 minutes [148]. McKeague et al. performed fifteen rounds of* in vitro* selection to identify ssDNA MREs specific for OTA. Two candidate MREs had reported *K*_*d*_ values of 110 ± 50 nM (designated B08) and 290 ± 150 nM (designated A08). A08 ssDNA MRE was utilized in a label-free fluorescence detection assay and achieved a LOD of 9 nM. It also had low cross-binding activity on OTB and warfarin. The authors reported a truncated version of A09 also had similar specificity and binding affinity profiles [149].

Fumonisins are heat-stable mycotoxins present in most corn and are produced by fungi,* Fusarium verticillioides* and* Fusarium proliferatum*. Fumonisin B_1_ (FB_1_) is a nephrotoxin and potential carcinogen in humans. As the toxin cannot be inactivated by cooking in high temperature, it is crucial to monitor its level during food production [252]. McKeague et al. performed eight rounds of selection to identify ssDNA MRE with high binding affinity toward FB_1_. Unmodified magnetic beads (immobilization substrate), L-homocysteine, L-cysteine, and L-methionine L-glutamic acid were used as negative targets in the selection. Six candidate MREs were identified, and the best candidate MRE had a reported *K*_*d*_ of 100 nM. However, the authors did not test the specificity of the selected MRE on other mycotoxins [13].

Zearalenone (ZEN; F-2 toxin) is a nonsteroidal estrogenic mycotoxin produced by many fungus species in the* Fusarium* genus. It has been shown to be present in many grains worldwide, such as oats, wheat, rice, and their derived food products [253]. Chen et al. performed fourteen rounds of selection, and the best candidate MRE had reported *K*_*d*_ of 41 ± 5 nM and high specificity. Cross-binding assays showed insignificant binding to other mycotoxins, *β*-zearalenol, aflatoxin B1, aflatoxin B2, fumonisin B_1_, and fumonisin B_2_. Circular dichroism measurement showed a conformational change of the MRE after binding of zearalenone. A detection assay using MRE immobilized magnetic beads and the blue-green florescence property of zearalenone was developed. A LOD of 0.785 nM was achieved in pretreated beer samples [150].

T-2 toxin (T-2) is a trichothecene mycotoxins produced by many species in the* Fusarium* genus and is harmful to humans. It is a very stable small molecule biological toxin that is resistant to high temperature and is present in variety of grains, such as oats, barley, and wheat. Currently, it can only be detected by labor intensive and costly instruments and it is thus difficult to monitor its level in food [254]. Chen et al. recently utilized ten rounds of GO-SELEX to identify ssDNA MRE specific for T-2 with high affinity and specificity. Fluorescent binding and cross-binding assay showed that the *K*_*d*_ of the best candidate MRE was in the nanomolar range, with insignificant cross-binding activities on FB_1_, ZEN, OTA, and aflatoxin B1. There was a conformational change upon MRE-T-2 binding. The authors also developed a fluorescent assay to detect spiked T-2 level in beer. A LOD of 0.4 *μ*M was achieved [151].

Aflatoxins are highly toxic natural compounds produced by many species of filamentous fungi and can contaminate agricultural products. The LD_50_ can be as low as 0.5 mg/kg, and acute toxicity is even higher than many chemical toxins, such as cyanide or arsenic [255, 256]. Ma et al. performed an* in vitro* selection on a subtype of aflatoxins, aflatoxins B1 (AFB1). After ten rounds of target and negative target selection, the best candidate MRE had a reported *K*_*d*_ of 11.39 ± 1.27 nM and with minimal cross-binding activities on aflatoxins B2, G1, G2, OTA, and FB_1_. A fluorescent assay similar to the authors' previous study on ZEN and T-2 specific MRE was developed to detect spiked levels of AFB1 in methanol-extracted peanut oil. The assay achieved a LOD of 35 ng/L [152]. Malhotra et al. perform two selections (SELEX1 and SELEX2) using slightly different methodologies to identify ssDNA MREs specific for both AFB1 and aflatoxins M1 (AFM1). In SELEX1, lambda exonuclease was used to generate ssDNA from amplified dsDNA. AFM1 coated magnetic beads were used as a positive target from round 1 to round 10, and AFB1 coated magnetic beads were used as positive target at round 11 (last round) only. Free targets were used to competitively elute ssDNA that bound to toxin coated beads in round 10 and round 11. In SELEX 2, each round started from preincubation with counter targets (uncoated beads, AFB1 beads) followed by incubation with AFM1 beads. Snap cooling was used to obtain ssDNA from dsDNA. In SELEX 2, only eight rounds were carried out. Multiple candidate MREs were analyzed and their *K*_*d*_ values were in the nano- to low micromolar range. One MRE with the best affinity (*K*_*d*_ = 35.6 ± 2.9 nM), designated AFAS3, was used in developing a colorimetric assay based on MRE immobilized gold nanoparticles. This assay had a detection range of 250 to 500 nM of AFM1 and only minor interaction with AFB1. However, there were no reported cross-binding data on other mycotoxins [153].

Two studies identified ssDNA MREs specific for biological toxins with therapeutic intentions. Alpha-Bungarotoxin is a toxic substance in krait snake venom and can bind irreversibly to acetylcholine receptors and eventually lead to death in victims [257, 258]. Lauridsen et al. performed a rapid one-step SELEX and identified ssDNA MRE with relatively high binding affinity toward Alpha-Bungarotoxin (*K*_*d*_ = 7.58 *μ*M). The authors claimed that rapid selection technique could potentially be used with a chemically modified nucleic acid library and generate MREs suitable for diagnostic and therapeutic purposes [31].

Vivekananda et al. selected ssDNA MRE specific for alpha-toxin of* Staphylococcus aureus*. Several candidate sequences showed cell rescuing effects when coadministrated with alpha toxin in multiple* in vitro* neutralization assays. The authors claimed that it was possible to generate MREs against alpha-toxin for the treatment of* S. aureus* infections [155].

Hong et al. also performed an* in vitro* selection against* S. aureus* alpha toxin. Twelve rounds of positive and eleven rounds of negative rounds of negative selection were performed to identify the candidate ssDNA MRE. The reported *K*_*d*_ determined by SPR single cycle kinetics was 93.7 ± 7 nM. Fluorescence plate based cross-binding assay showed the ssDNA MRE was approximately two to five times more selective on the alpha toxin than negative targets. A proof-of-concept modified ELISA using the selected ssDNA MRE had a reported sensitive target detection at 200 nM in human serum [154].

#### 2.5.2. Chemical Toxins

The detection of chemical toxins is important in both food safety and environmental monitoring. Environmental and food contamination by various kinds of chemical toxins have been reported and even at low concentrations can still be detrimental to human health. Currently, the majority of small chemical toxins can only be detected by labor intensive and costly laboratory equipment such as liquid and/or gas chromatography coupled with mass spectrometry. In order to address these current limitations, there has been an increase in the identification and biosensing applications of MREs with high affinity and specificity to capture and detect chemical toxins. However, the* in vitro* selection of ssDNA MREs targeting small molecule chemical toxins has several inherent challenges, such as difficulties in efficient separation between bound and unbound DNA molecules, limited chemical motifs on target surfaces for sufficient binding, lack of chemical functional groups for target immobilization, and candidate MREs that may not have sufficient specificities to distinguish molecules with very similar chemical structures if selection schemes are not carefully designed. For these reasons, there are a limited number of ssDNA MREs specific for chemical toxins currently in the literature (Table 5).

Kim et al. identified a 76-mer ssDNA MRE specific for 17-beta-estradiol (E2) with a *K*_*d*_ of 0.13 *μ*M after seven rounds of selection. Specificity of the selected MRE was shown by square wave voltammetry (SWV) measurement, with only minimal binding to structurally similar organic chemicals 2-methoxynaphthalene and 1-aminoanthraquinone. The authors initially attempted SPR for the detection of E2. However, due to the small molecular weight of E2, there were no observable binding events by SPR. An electrochemical platform measured under SWV was eventually utilized to detect E2 with a LOD of 0.1 nM in buffer solutions [156]. Alsager et al. selected a 75-mer ssDNA MRE specific for E2 with a *K*_*d*_ of 50 nM after eighteen rounds of selection. The 5′ amino-modified MRE was covalently conjugated to carboxylated nanoparticles and dynamic light scattering/resistive pulse sensing was used to observe size contraction in particle size upon E2 binding. A detection range of 5 nM to 100 nM was achieved with this detection platform. Progesterone, testosterone, Bis (4-hydroxyphenyl) methane (BPF), and bisphenol-A (BPA) were also tested for the specificity of the selected MRE. The assay showed minimal binding to both BPA and BPF; however, the MRE was not able to distinguish the other two steroids [157].

Bisphenol A (BPA) is an estrogen mimicking chemical that has been classified as an endocrine-disrupting compound. It is used in the manufacture of polycarbonate plastic products, such as plastic bottles and containers. It has been shown to be released into food after heating and can accumulate in human [259]. Jo et al. selected ssDNA MRE specific for Bisphenol A with high affinity and specificity. The reported *K*_*d*_ was 8.3 nM with only minimal binding to structurally related chemical molecules, including 6F biophenol A, bisphenol B, and 4, 4′-bisphenol. A cy-3 labeled MRE pair was immobilized on sol-gel biochip and a sandwich detection assay was developed with nanomolar range sensitivity. However, the authors acknowledged the assay system can only detect a limited range of BPA concentrations [158].

Polychlorinated biphenyls (PCB) are a group of chlorinated hydrocarbons that are used in varies of industrial settings. PCBs are highly toxic and are reported to be an environmental contaminant affecting water bodies and food sources [260]. Mehta et al. identified PCB binding ssDNA MREs with nanomolar range affinity. In their study, two PCB compounds with hydroxyl functional group were immobilized on magnetic beads and used as target for selection. After nine rounds of selection, three candidate sequences were chosen for characterization. Two of the three candidate sequences (9.1 and 9.3) showed comparable binding affinities to both immobilized targets. In subsequent crossing-binding analysis, candidate 9.1 showed broad substrate binding affinity to other PCB compounds, while candidate 9.2 showed a high specificity for the two PCBs with hydroxyl functional groups. The study did not test specificity on other hydrocarbons that are structurally similar to PCB [160]. Xu et al. immobilized a primary amine modified PCB compound (PCB77-NH_2_) on epoxy-activated Sepharose agarose as the target for* in vitro* selection. After 11 rounds of selection, four candidate sequences were characterized to have affinity in the low micromolar range. Cross-binding assays showed only minimal binding toward other hydrocarbons and agarose substrate. A fluorescent based detection assay was developed using the fluorescence quenching property of gold nanoparticle. Upon binding to target, the fluorescent signal was released. A detection range of 0.1–100 ng/mL was achieved. This assay detected other PCB compounds with different sensitivities [159].

The current detection method for herbicides and pesticides environmental contaminants in the environment relies on using time-consuming and labor intensive laboratory based equipment. MREs have been investigated as binding elements in rapid, field deployable detection systems. Atrazine is a widely used herbicide worldwide [261]. Sanchez utilized CE-SELEX to identify ssDNA MRE specific for atrazine with a *K*_*d*_ of 890 nM. However, the MRE did not show specificity in binding between atrazine and structurally closely related simazine at concentration below approximately 2 *μ*M in a fluorescence polarization detection assay [161].

Williams et al. also performed an* in vitro* selection of ssDNA MRE specific for atrazine. A derivative of atrazine, desethyl-atrazine was first biotinylated and then immobilized on streptavidin coated magnetic beads. The selection scheme was designed with increasing selection stringency, by incorporating negative selections on streptavidin magnetic beads, simazine, metabolites of atrazine, and other commonly used pesticides. Competition selection was also performed to ensure the library bound only to free atrazine in solution, but not to desethyl atrazine. As a result, ssDNA MRE with subnanomolar affinity and high specificity was identified after twelve rounds of selection. A magnetic bead based capture assay coupled with capillary electrophoresis was developed to detect atrazine in artificially contaminated river water samples. The assay was able to detect atrazine in the nanomolar range [14]. Similar* in vitro* selection methodology was also employed by Williams et al. to identify MREs specific for a commonly used organophosphate pesticide, malathion. In their second selection, the selected MRE had high nanomolar range affinity, and minimal binding to metabolites of malathion and other herbicides. However, the author noted that the cross-binding activity was high on bovine serum albumin possibility due to the large, globular characteristics of the protein [15]. William et al. subsequently performed another selection on an herbicide, bromacil. This study further validated the methodology the authors employed to identify MREs with high affinity and low cross-binding activities on structurally similar compounds and compounds that were likely to coexist in the environment. The authors noted that these properties were particularly important for incorporating ssDNA MREs as sensing elements in biosensors [16].

As noted above, not every chemical toxin can be readily immobilized for portioning during selection. In order to circumvent this limitation, Wang et al. utilized an immobilization free* in vitro* SELEX developed by Li and coworkers to select ssDNA MREs specific for four different organophosphorus pesticides, phorate, profenofos, isocarbophos, and omethoate [26, 28]. After twelve rounds of selection, two candidate sequences reported *K*_*d*_ values in the low micromolar range for all four targets. Cross-binding assays showed good specificities for the selected two MREs, with only minimal observed binding to eight other different pesticides [28]. The same group of researchers later developed a fluorescence polarization assay using the selected MREs to detect phorate, profenofos, isocarbophos, and omethoate at a LOD of 19.2, 13.4, 17.2, and 23.4 nM, respectively [247].

He et al. employed immobilization-free SELEX to identify ssDNA MRE specific for pesticide, acetamiprid. After eighteen rounds of selection, the best candidate MRE was reported to have a *K*_*d*_ of 4.96 *μ*M. Specificity of the selected MRE was tested and cross-binding data showed no significant change in fluorescent signals in the presence of three other pesticides, imidacloprid, nitenpyram, and chlorpyrifos. The authors noted that the affinity of the selected MRE was lower than typical antibodies [27].

GO-SELEX was used to identify three ssDNA MREs specific to three different pesticides: tebuconazole, mefenacet, and inabenfide [25]. The reported values of *K*_*d*_ were in the range of 10 to 100 nM. High specificity of each identified MRE was also determined by isothermal titration calorimetric and gold nanoparticle colorimetric assays. A simple, rapid detection method using gold nanoparticles was developed with LOD ranges from 100 to 400 nM.

## 3. General Classes of Detection Methods

In recent years, a large number of researches have taken place in applying ssDNA MREs for the use in biosensors. Major detection methods can be categorized into three classes: (1) electrical/electrochemical, (2) optical, and (3) mass sensitive. The following section highlights the basic principles of the general classes of detection methods that have been utilized widely in the development of ssDNA MRE based biosensors. The relative portability of different detection methods is also briefly discussed. Recent literatures describing the detection of pathogens and toxins using ssDNA MREs biosensors are summarized in Tables 6, 7, and 8.

### 3.1. Electrical/Electrochemical

**Figure 2:**
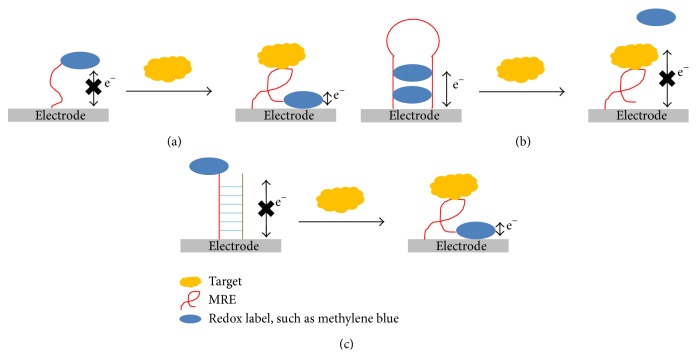
Illustration of examples of ssDNA MRE based electrochemical biosensors. (a) A representation of an “on-mode” system using a redox label for current transduction. (b) A representation of a “label-free” system by intercalating a redox label in a hairpin structure. (c) A representation of an “on-mode” system by hybridization with the complementary sequence.

The principle of electrochemical detection is based on measuring changes in electrical properties of the sensing platform. In this method, ssDNA MRE is usually immobilized on a gold electrode via thiol-gold linkage. A redox label, such as methylene blue, can be used to detect binding between MRE and the target [262]. In a “signal on” system, the redox label is away from the electrode surface, and the binding of target causes a conformational change in the MRE and brings the redox label into close proximity with the electrode, thus causing a measurable change in electrical properties (Figure 2). A “signal off” system behaves similarly, but the binding of target causes the redox label to move away from the electrode. This system can also be modified as a “label-free” system, in which the redox molecule is intercalated in a hairpin structure of a MRE in a target unbound state, and binding of the target causes the release of the redox molecule (Figure 2). In addition to measuring redox current, the changes in impedance upon binding of the target can also be measured. In this case, no labeling of MRE is required and the conformational changes in MRE upon target binding cause a measurable change in impedance that can be recorded by voltammetry [212].

**Figure 3:**
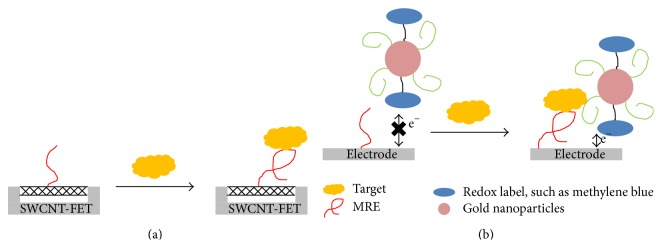
Illustration of examples of signal enhancement methods in ssDNA MRE based electrochemical biosensors. (a) A representation of a single-walled carbon nanotubes field effect transistors. (b) A representation of gold nanoparticles carrying redox labels in a sandwich detection style.

Nanomaterials can also be incorporated into electrochemical sensor to enhance signals. Single-walled carbon nanotube field effect transistors (SWCNT-FET) can be used to build electrochemical biosensors (Figure 3). In this system, MREs are immobilized on SWCNTs and SWCNTs are sandwiched between a source and a drain electrode. When the immobilized MREs bind to the target, there is a measurable change in the conductance of the system [240]. Gold nanoparticles (AuNP) are also widely used as signal enhancers. AuNPs can be coated on electrodes and greatly increase the surface area. As a result, more MREs can be immobilized on the electrode, thus enhancing the system's sensitivity. AuNPs can also be coated with a second MRE and reporting probes in a sandwich assay (Figure 3). In this case, the target first binds to a primary capturing MRE, followed by the binding a secondary reporting MRE along with a redox molecule, which can generate an enhanced signal for sensitive detection [263].

In general, electrical/electrochemical detection systems are relatively smaller and more easily adapted into portable, chip-based platforms. This allows the ssDNA MREs biosensors to be used for on-site target detection [211, 212].

### 3.2. Optical

Optical detection methods can be classified into three major categories: (1) fluorescence detection, which require specialized instruments to measure fluorescent signals; (2) colorimetric detection, which color changes can be observed by the naked eye or measured in terms of optical density; (3) absorbance assay can enhance detection signals, and subsequently be measured by instruments as well.

#### 3.2.1. Fluorescence

**Figure 4:**
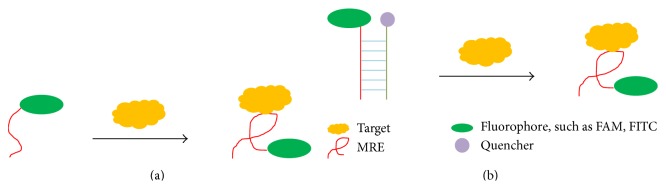
Illustration of examples of ssDNA MRE based fluorescent biosensors. (a) A representation of the changes in fluorescent signal upon target binding to a fluorophore labeled MRE. (b) A representation of an “on-mode” system by using a quencher labeled on the complementary sequence.

The principle of fluorescence detection is based on the generation or quenching of fluorescence signals upon target binding. Various fluorescence molecules and quantum dots can be linked to ssDNA MREs. Conformational changes induced by target binding can alter the fluorescence signal generated by the fluorophore and therefore can be measured (Figure 4) [264]. Quenching molecules can also be linked to the other end of the ssDNA MRE. In this system, the quencher completely blocks the fluorescence signal from the fluorophore and target binding can move the quencher away from the fluorophore and have “signal on” detection (Figure 4) [265]. The same principle can also be applied for a “signal off” system. Carbon nanotubes and graphene can also be used as quenchers, where fluorescent labeled ssDNA MREs is adsorbed on the carbon quenchers via *π*-*π* stacking interactions. Fluorescence resonance energy transfer (FRET) can also be utilized as measurements when the distance of the two fluorescence molecules linked to MREs is changed upon target binding.

Fluorescence based detection systems are frequently developed because of their high sensitivity and the ease of fluorescently labeling ssDNA MREs. Traditionally, the complete detection system requires large, costly components, including lasers, filters, and detectors. A recent study reported a portable ssDNA MRE based biosensor utilizing an optic fiber for sensitive detection of BPA [233].

#### 3.2.2. Colorimetric

**Figure 5:**
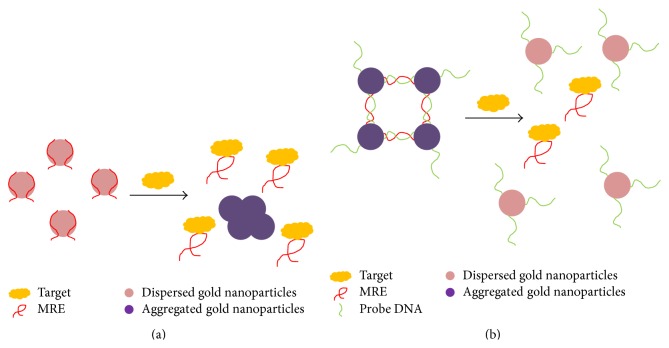
Illustration of examples of ssDNA MRE based colorimetric biosensors. (a) A representation of a colorimetric assay using MRE dispersed gold nanoparticles. (b) A representation of a colorimetric assay using cross-linked gold nanoparticles aggregates via MRE and probe DNA.

Gold nanoparticles (AuNP) have been widely used in various colorimetric assays. AuNPs aggregate in salt solution and appear in purple color. When they are dispersed, they are in red color. This special absorbance property of AuNPs allows observation of target binding by naked eye. MREs in salt solution can bind to AuNPs, dispersing the AuNPs. When targets are introduced into the system, MRE preferably bind to the targets, therefore causing AuNPs to aggregate, and a red to purple color change is observed (Figure 5) [266]. Alternatively, ssDNA MREs can be used to link AuNPs that are functionalized with probe strands. In this case, the initial state of the MRE/AuNPs solution is aggregated purple. Upon target binding, the linked AuNPs are released, and a purple to red color change is observed (Figure 5) [267]. Furthermore, AuNPs can be used in a sandwich colorimetric assay, in which the secondary reporting MRE linked AuNP can grow in size when the detection system is placed in a growth solution containing HAuCl_4_, thus enhancing the detection limit [268].

Colorimetric detection systems are attractive for on-site target sensing due to the ease of observation with the naked eye. These systems are often developed into hand-held laminar flow devices [163, 204, 226].

#### 3.2.3. Absorbance

**Figure 6:**
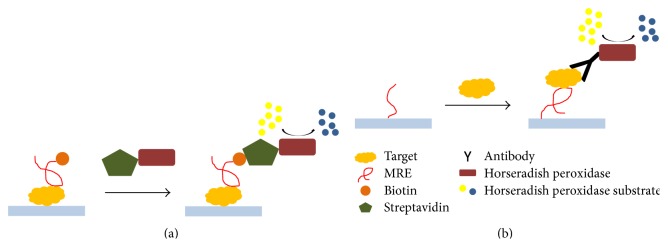
Illustration of examples of ssDNA MRE modified enzyme linked assays. (a) A representation of a direct MREs modified enzyme linked assay with MRE as the reporter. (b) A representation of an indirect MREs modified enzyme linked assay with MRE as the target capturing element.

Nucleic acid MREs have been used in modified enzyme linked immunoassays, usually substituting for either the capturing or the reporter antibodies. In a direct oligonucleotide enzyme link assay, often the protein target is adsorbed on plate and biotinylated MREs bind to the target and then followed by the addition of streptavidin-horse radish peroxidase (HRP) conjugate and enzyme substrate for signal development [148]. In a sandwich assay, biotinylated MREs can be immobilized on streptavidin plate and then followed by the addition of the protein target, HRP linked antibody, and enzyme substrate [98]. This detection method is mostly limited to clinical laboratory settings and detection of protein targets for which antibodies have been isolated (Figure 6).

#### 3.2.4. Mass Sensitive

Mass sensitive detection is a class of label-free detection system that can be subdivided into four major categories: (1) surface plasmon resonance (SPR), (2) quartz crystal microbalance (QCM), (3) surface acoustic wave (SAW), and (4) micromechanical cantilever. None of these detection systems require additional labeling.

**Figure 7:**
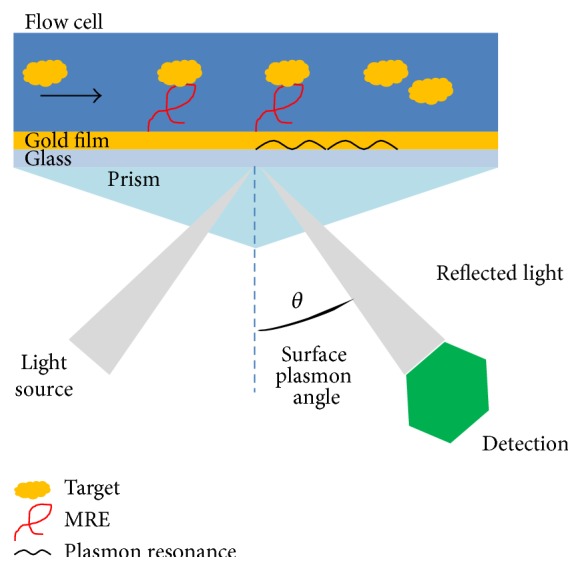
Illustration of ssDNA MRE based surface plasmon resonance biosensors. When targets bind to immobilized MREs, a change in the plasmon resonance and plasmon angle will be detected and translated into a real-time response unit.

Surface plasmon resonance (SPR) sensors measure a change in the refractive index and resonance angle when a mass change occurs upon target binding. MREs are often biotin-tagged and immobilized on streptavidin coated gold chip. When targets in solution pass through the flow cell, the binding between targets and immobilized MREs cause a change in mass on the sensor chip surface and is subsequently translated into a change in the refractive index. This change in resonance is proportional to the amount of target bound to the immobilized MREs and therefore providing a real-time detection of the target in solution (Figure 7) [183]. Commercially available SPR systems are typically large and limited to bench top use. One study reported a portable SPR biosensor based on ssDNA MREs for the detection of H5N1 influenza virus [183].

**Figure 8:**
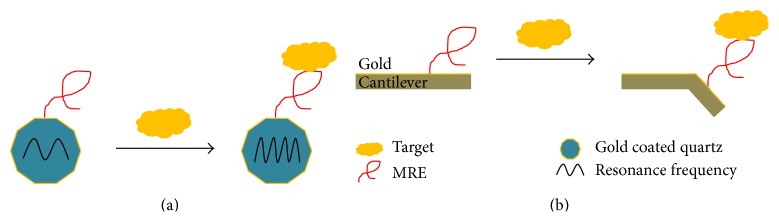
Illustration of examples of ssDNA MRE based mass sensitive biosensors. (a) A representation of a detectable change in resonance frequency upon target binding to immobilized MRE on quartz crystal microbalance. (b) Representation of a detectable nanometer scale bending upon target binding to immobilized MRE on micromechanical cantilever.

A Quartz crystal microbalance (QCM) is an acoustic wave resonator based on the piezoelectric property of quartz crystal. Nucleic acid MREs can be immobilized on gold-coated quartz. The binding between target and MRE increases the mass on the surface of the crystal and leads to a detectable decrease in the resonance frequency of the crystal (Figure 8) [181]. The detection principle of surface acoustic wave (SAW) based biosensor is similar to QCM. Nucleic acid MREs have been utilized to fabricate a special type of Love-wave sensor that uses shear horizontal waves to enhance the surface sensitivity and achieve ultrasensitive detection of the target [269].

Micromechanical cantilevers have been investigated for MRE based biosensors. The major advantage of this type of sensor is that it can be readily scale up and perform parallel analysis for multiple analytes with low background interference [270]. When the target binds to the MRE on the surface of the cantilever, a nanometer scale deflection in the cantilever can be detected optically (Figure 8). Zhao et al. reported the development of a ssDNA MRE based piezoresistive microcantilever biosensor for potential on-site detection of Staphylococcal enterotoxin B [188].

## 4. Conclusions and Future Perspectives

Over the last two decades, there has been a continuous increase in the research of molecular recognition elements. Single-stranded DNA MREs have several advantages over antibodies, in terms of stability, reusability, and production cost. However, ssDNA MREs are not without limitations. The binding affinity of MREs is highly dependent on their three-dimensional structure and is influenced by factors including the ionic condition, temperature, and pH of the binding condition [4]. Challenges remain in eliminating cross-binding activities to other molecules in native environments. These limitations hinder the use of MRE for detection in many real world complex samples, such as biological fluids and food matrices. A carefully designed selection scheme can greatly improve the specificity of the identified MRE, which can better distinguish closely related molecules at low concentrations. Using modified bases in PCR amplification or performing base modifications after selection can also help improving resistance to nucleases in many biological fluids, such as human serum [271].

As the field of ssDNA MRE based biosensors continues to grow, improvements in SELEX methodology will be necessary to more rapidly isolate MREs with the desired affinity and specificity. Improvements will also be necessary to allow MREs against more targets and a wider variety of targets to be isolated. The development of MRE based sensors is becoming an increasingly diverse field. Scientist and engineers from many disciplines must work together in order to create the optimal end product. Portable ssDNA MRE based biosensors may be utilized in a variety of settings, such as food safety, environmental monitoring, and health care. The many attractive features of ssDNA MREs prompt researchers to continue to investigate and optimize their applications in biosensing. The commercialization of these devices should continue to increase in the future.

## Figures and Tables

**Table 1:** Summary table of ssDNA MREs targeting bacteria and bacteria structural components.

Target	SELEX method	*K*_*d*_	Detection method	LOD	Reference
*E. coli *K88	Cell-SELEX	15 ± 4 nM	Fluorescence	1100 CFU/mL	[40]

*E. coli *	Cell-SELEX	12.4 to 25.2 nM	—	—	[42]

*E. coli *NSM59	Cell-SELEX	110 nM	—	—	[43]

*E. coli *K88 fimbriae protein	Plate immobilized	25 ± 4 nM	—	—	[41]

*E. coli *8739 outer membrane protein	Magnetic beads	—	FRET	30 CFU/mL	[44]

*E. coli *O111:B4 Lipopolysaccharide	Magnetic beads	—	—	—	[45]

*Salmonella *Typhimurium	Cell-SELEX	1.73 ± 0.54 *µ*M	Magnetic capture-qPCR	100–1000 CFU	[46]

*Salmonella *Typhimurium	Cell-SELEX	6.33 ± 0.58 nM	Fluorescence	25 CFU/mL	[47]

*Salmonella *Typhimurium	Cell-SELEX	—	—	—	[48]

*Salmonella *Typhimurium outer membrane protein	Nitrocellulose membrane	—	Magnetic capture-qPCR (spike and recovery)	<10 CFU/g	[49]

*Salmonellae *Typhimurium/enteritidis	Cell-SELEX	Nanomolar to micromolar range	—	—	[51]

*Salmonellae *enteritidis/Typhimurium	Cell-SELEX	7 nM, 25 nM	—	—	[50]

*Salmonella *Paratyphi A	Cell-SELEX	47 ± 3 nM	Chemiluminescence	1000 CFU/mL	[52]

*Salmonella *O8	Cell-SELEX	32.04 nM	—	—	[53]

*Vibrio alginolyticus *	Cell-SELEX	27.5 ± 9.2 nM	PCR	100 CFU/mL	[56]

*Vibrio parahaemolyticus *	Cell-SELEX	16.88 ± 1.92 nM	—	—	[55]

*Listeria monocytogenes *	Cell-SELEX	Midnanomolar range	—	—	[57]

*Listeria monocytogenes *	Cell-SELEX	35.7 ± 8.02 *µ*M	Magnetic capture-qPCR	<60 CFU/500 *µ*L	[58]

*Listeria monocytogenes *	Cell-SELEX	60.01 nM	Fluorescence	—	[60]

*Listeria monocytogenes *	Cell-SELEX	48.74 ± 3.11 nM	Fluorescence	75 CFU/mL	[59]

*Listeria monocytogenes* Internalin A	Filter plate	—	Fiber optic	1000 CFU/mL	[62]

*Shigella dysenteriae *	Cell-SELEX	23.47 ± 2.48 nM	Fluorescence	50 CFU/mL	[64]

*Streptococcus mutans *	Cell-SELEX	33 nM	Colorimetric (flow-through)	10^5^–10^8^ CFU/mL	[70]

*Streptococcus pyogenes *	Cell-SELEX	9, 10 nM	—	—	[72]

*Staphylococcus aureus *	Cell-SELEX	35, 129 nM	Optical light scattering	1 CFU/mL	[75]

*Staphylococcus aureus *	Cell-SELEX	Nanomolar range	—	—	[74]

*Proteus mirabilis *	Cell-SELEX	7.7 nM, 4.1 nM	—	—	[69]

*Pseudomonas aeruginosa *	Cell-SELEX	Low nanomolar range	Fluorescence	—	[78]

*Mycobacterium tuberculosis *	Cell-SELEX	*K*_*a*_ 10^5^–10^6^ M	—	—	[80]

*Francisella tularensis *subspecies (subsp.)* japonica *bacterial antigen	Cell-SELEX	—	ALISA	1700 bacteria/mL	[86]

*Bacillus anthracis *spores/anthrose sugar	Magnetic beads	—		30,000 spores/mL	[84]

*Bacillus anthracis *spores	Magnetic beads	—	Magnetic bead-electrochemiluminescence	10.6 × 10^6^ spores	[82]

*Bacillus thuringiensis *spores	Magnetic beads	—	Fluorescence	1000 CFU/mL	[83]

*Campylobacter jejuni *	Cell-SELEX	292.8 ± 53.1 nM	—	—	[68]

*Campylobacter jejuni* (surface protein)	Magnetic beads	—	Fluorescence (magnetic bead/quantum dot)	10–250 CFU in food matrix, 2.5 CFU in buffer	[66]

*Campylobacter jejuni* (killed)	CE-SELEX	—	Capillary electrophoresis	6.4 × 10^6^ cells/mL	[67]

Peptidoglycan	Filter	0.415 ± 0.047 *μ*M/1.261 ± 0.280 *μ*M	—	—	[88]

Lipopolysaccharide (endotoxin)	NECEEM non-SELEX	Low to high nanomolar range	Electrochemical	0.01–1 ng/mL	[91]

**Table 2:** Summary table of ssDNA MREs targeting viruses and virus protein for biosensing applications.

Target	SELEX method	*K*_*d*_	Detection method	LOD	Reference
Human noroviruses	Antibody-bead conjugates	High nanomolar range	RT-qPCR	10 RNA copies	[94]

Norovirus	Nitrocellulose membrane	Low picomolar range	Electrochemical	180 virus particles	[93]

Norovirus II.4 capsid protein VP1	Filter	—	—	—	[95]

Influenza A H1N1	Microfluidic SELEX	55.14 ± 22.40 nM	Bead/fluorescent	6.4 × 10^−3^ HAU	[34]

Avian influenza H5N1	Nitrocellulose membrane	4.65 nM	Dot blot	1.28 HAU	[97]

Influenza A hemagglutinin protein	TALON affinity resin	Low nanomolar range	Sandwich ELAA	—	[98]

SARS-CoV N protein	Ni-NTA beads	4.93 ± 0.3 nM	Western blot	—	[10]

Bovine viral diarrhea virus	GO-SELEX	5 × 10^4^ TCID_50_/mL	SPR AuNP sandwich	800 copies/mL	[24]

HCV envelope surface glycoprotein E2	Cell surface SELEX	1.05 ± 1 nM	—	—	[100]

Dengue virus type-2 envelope protein domain III	Ni-NTA magnetic beads	154 ± 40 nM	—	—	[102]

HIV reverse transcriptase	CE-SELEX	180 ± 70 pM	—	—	[103]

**Table 3:** Summary table of ssDNA MREs targeting viruses and virus proteins for therapeutic applications.

Virus	Target	SELEX method	*K*_*d*_	Reference
HIV	Reverse transcriptase	Nitrocellulose filters	—	[126]

HIV	Reverse transcriptase	Sephadex columns	660 pM	[127]

HIV	Reverse transcriptase	Nitrocellulose filters	1 nM	[128]

HIV	Reverse transcriptase	Primer-free SELEX	82 nM	[129]

HIV	Integrase	Nitrocellulose filters	—	[130]

HIV	Integrase	—	—	[131]

HIV	Trans-activation-responsive RNA (TAR) element	Magnetic beads	20 nM	[132]

HIV	Trans-activation-responsive RNA (TAR) element	Magnetic beads	50 nM	[133]

HCV	NS5B RNA polymerase	Nitrocellulose filters	132 nM	[134]

HBV	Core protein	—	High affinity determined by dot blot	[135]

SARS coronavirus	Helicase	Magnetic beads	5 nM	[136]

Influenza A virus	Hemagglutinin from H3N2	Ni-NTA beads	—	[137]

Influenza A virus	Hemagglutinin from H5N1	Ni-NTA beads	High affinity determined by ELISA	[138]

Influenza A virus	Hemagglutinin from H3N2	Ni-NTA magnetic beads	7 nM	[139]

Influenza A virus	Hemagglutinin from H9N2	Nitrocellulose filters	High affinity determined by ELISA	[140]

Influenza A virus	Nonstructural protein 1	Glutathione agarose beads	18.91 ± 3.95 nM	[141]

Rabies virus	Rabies virus infected BHK-21 cells	Cell-SELEX	28 nM	[142]

HPV	HPV transformed HeLa cells	Cell-SELEX	1 nM	[143]

Vaccinia virus	Whole virus particle	One-step MonoLEX	High affinity determined by dot blot, SPR, and so forth	[144]

**Table 4:** Summary table of ssDNA MRE targeting biological toxins and virulence factors.

Target	SELEX method	*K*_*d*_	Detection method	LOD	Reference
Enterotoxin B	Magnetic beads	—	Electrochemiluminescence	10 pg	[105]

Enterotoxin B	Magnetic beads	—	—	—	[11]

Enterotoxin C1	Magnetic beads	65.14 ± 11.64 nM	Fluorescence	6 ng/mL	[106]

Cholera toxin	Magnetic beads	—	ELAA/electrochemiluminescence	10 ng 40 ng	[105]

*C. difficile *toxin A/toxin B/binary toxin	Magnetic Beads/SOMAmer	Sub- to low nanomolar range	Various	1 pmol/L	[109]

*C. difficile *binary toxin	Sandwich SELEX/SOMAmer	0.02–2.7 nM	Sandwich assays	Low picomolar	[110]

*C. difficile *toxin B	Magnetic beads	47.3 ± 13.7 nM	Modified ELISA	50 nM	[111]

CFP-10.ESAT-6 heterodimer	Nitrocellulose	Low nanomolar range	Colorimetric (ELONA)	100% sensitivity, 68.75% specificity	[9]

CFP-10.ESAT-6 heterodimer	Microwell plate	375 nM/160 nM	Colorimetric (ELONA)	89.6–100% sensitivity, 94.1% specificity	[115]

MPT64 TB protein	Microwell plate	—	Sandwich assays	—	[117]

Protective antigen	CE-SELEX	112 nM	Electrochemical (SWNT)	1 nM	[119]

Protective antigen	Membrane filtration	Nanomolar range	ELISA	—	[120]

Botulinum neurotoxin type A heavy chain peptide/toxoid	Single microbead	Nano- to micromolar range	—	—	[30]

Botulinum neurotoxin type A light chain	Microfluidic-SELEX	Low nanomolar range	—	—	[32]

Botulinum neurotoxin type A light chain	Magnetic beads	—	Fluorescence	1 ng/mL	[122]

Microcystin	Sepharose gel	*K*_*a*_: 10^3^ M^−1^	SPR	—	[123]

Cylindrospermopsin	Sepharose beads	88.78 nM	Electrochemical	100 pM	[125]

Saxitoxin	Magnetic beads	—	—	—	[145]

Okadaic acid	Magnetic beads	77 nM	Electrochemical	70 pg/mL	[12]

Ochratoxin A	Agarose resin	200 nM	Fluorescence polarization	5 nM	[146, 147]

Ochratoxin A	Magnetic beads	96–293 nM	ELAA	1 ng/mL	[148]

Ochratoxin A	Sepharose beads	High nanomolar range	Fluorescence	9 nM	[149]

Fumonisin B_1_	Magnetic beads	100 nM	—	—	[13]

Zearalenone	Magnetic beads	41 ± 5 nM	Fluorescence	0.785 nM	[150]

T-2 toxin	GO-SELEX	20.8 ± 3.1 nM	Fluorescence	0.4 *µ*M	[151]

Aflatoxin B1	Magnetic beads	11.39 ± 1.27 nM	Fluorescence	35 ng/L	[152]

Aflatoxin B1/M1	Magnetic beads	96–221 nM 35–1515 nM	Colorimetric/AuNPs	250–500 nM (Aflatoxin M1)	[153]

Alpha-bungarotoxin	1 step SELEX	7.58 *µ*M	—	—	[31]

Alpha toxin	Magnetic beads	93.7 ± 7 nM	Modified ELISA	200 nM	[154]

Alpha toxin	Filter	—	—	—	[155]

**Table 5:** Summary table of ssDNA MREs targeting chemical toxins.

Target	SELEX method	*K*_*d*_	Detection method	LOD	Reference
17*β*-estradiol (E2)	Sepharose column	0.13 *µ*M	Electrochemical	0.1 nM	[156]

17*β*-estradiol (E2)	Sepharose column	50 nM	Dynamic light scattering, resistive pulse sensing	5 nM–100 nM	[157]

Bisphenol A	Epoxy-activated resin	8.3 nM	Fluorescence	Nanomolar range	[158]

Polychlorinated biphenyls	FluMag-SELEX	Low micromolar range	Fluorescence	0.1 to 100 ng/mL	[159]

Polychlorinated biphenyls	Magnetic beads	Nanomolar range	**—**	**—**	[160]

Atrazine	CE-SELEX	890 nM	Fluorescence polarization	**—**	[161]

Atrazine	Magnetic beads	0.62 ± 0.21 nM	Magnetic beads capturing coupled with CE	Nanomolar range	[14]

Malathion	Magnetic beads	1.14 ± 0.7 nM	**—**	**—**	[15]

Bromacil	Magnetic beads	9.6 ± 7.8 nM	**—**	**—**	[16]

Tebuconazole Mefenacet Inabenfide	GO-SELEX	10–100 nM	Colorimetric	100–400 nM	[25]

4 organophosphorus pesticides phorate, profenofos, isocarbophos, and omethoate	Filtration column/immobilization free	Low micromolar range	**—**	**—**	[28]

Acetamiprid	Immobilization free	4.98 *µ*M	**—**	**—**	[27]

**Table 6:** Summary table of ssDNA MRE based biosensors for the detection of pathogens.

Target	Detection methods	Enhancers	LOD	Relevant sample	Reference
*Salmonella *enteritidis	Fluorescence	Graphene oxide	40 CFU/mL	Milk	[162]

*Salmonella *enteritidis	Colorimetric lateral flow	Gold nanoparticles	10 CFU/mL	Milk powder	[163]

*Salmonella *Typhimurium	Colorimetric	Silver staining	7 CFU/mL	Lake water	[164]

*Salmonella *Typhimurium	Electrochemical impedance	Graphene oxide, gold nanoparticles	3 CFU/mL	Pork	[165]

*Salmonella *Typhimurium	Electrochemical impedance	Gold nanoparticles	600 CFU/mL	—	[166]

*Salmonella *Typhimurium	Colorimetric enzyme linked assay	Gold nanoparticles	1000 CFU/mL	Milk	[167]

*Vibrio parahaemolyticus*/*Salmonella *Typhimurium	Fluorescence flow cytometry	Quantum dots	5000 CFU/mL	Shrimp	[168]

*Salmonella *Typhimurium/*Staphylococcus aureus *	Fluorescence	—	5 CFU/mL 8 CFU/mL	—	[169]

*Staphylococcus aureus *	Electrochemical impedance	Single-walled carbon nanotubes	800 CFU/mL	Pig skin	[170]

*Staphylococcus aureus *	Electrochemical impedance	Graphene oxide	1 CFU/mL	—	[171]

*Staphylococcus aureus *	Colorimetric	Horseradish peroxidase	9 CFU/mL	Milk	[172]

Group A *Streptococcus *	Piezoelectric quartz crystal	Single-walled carbon nanotubes	12 CFU/mL	Milk	[173]

*E. coli *	Electrochemical/fluorescence	MRE cocktails	370 CFU/mL	—	[174]

*E. coli* O157:H7/*Salmonella *Typhimurium	Colorimetric	—	10000 CFU/mL	—	[175]

*E. coli* O111	Electrochemical redox current	Alkaline phosphatase	305 CFU/mL	Milk	[176]

*E. coli* CECT 675	Electrochemical impedance	Single-walled carbon nanotubes	6 CFU/mL 26 CFU/mL	Milk, apple juice	[177]

*Pseudomonas aeruginosa *	Fluorescence	—	5 CFU/mL	Drinking water	[178]

*Staphylococcus aureus*/*Vibrio parahaemolyticus*/*Salmonella* Typhimurium	Luminescence	Lanthanide-doped near infrared to visible upconversion nanoparticles	25 CFU/mL 10 CFU/mL 15 CFU/mL	Milk, shrimp	[179]

*Lactobacillus acidophilus*/*Staphylococcus enteric*/*Staphylococcus aureus *	Fluorescence microfluidic biochip	—	11 CFU/mL 61 CFU/mL 800 CFU/mL	—	[180]

Influenza H5N1	Quartz crystal microbalance	Hydrogel	0.0128 HAU	—	[181]

Vaccinia virus	Electrochemical impedance	—	60 virions/*µ*L	—	[182]

Influenza H5N1	Surface plasmon resonance	—	0.128 HAU	Poultry	[183]

**Table 7:** Summary table of ssDNA MRE based biosensors for the detection of biological toxins.

Target	Detection methods	Enhancers	LOD	Relevant sample	Reference
Prion protein	Resonance light scattering	Gold nanoparticles	0.01 nM	Human serum	[184]

*Clostridium difficile* Toxin A	Electrochemical impedance	Horseradish peroxidase	1 nM	—	[185]

Staphylococcal enterotoxin B	Electrochemical impedance	Horseradish peroxidase	0.24 ng/mL	Human serum	[186]

Staphylococcal enterotoxin B	Surface-enhanced Raman scattering	Gold nanoparticles	224 aM	Milk, blood, urine	[187]

Staphylococcal enterotoxin B	Piezoresistive microcantilevers	—	6 ng/mL	Skim milk	[188]

*E. coli* outer membranes proteins	Evanescent wave fiber optic	—	0.1 nM	Environmental water	[189]

*E. coli* outer membranes proteins	Electrochemical impedance	—	100 nM	Environmental water	[190]

*Botulinum* neurotoxin, type A	Electrochemical redox current	Horseradish peroxidase	40 pg/mL	—	[191]

Aflatoxin B1	RT-qPCR	—	25 fg/mL	Chinese wild rye hay, infant rice cereal	[192]

Aflatoxin B1	Fluorescence dipstick	—	0.3 ng/g	Corn	[193]

Aflatoxin M1	Electrochemical redox current	Magnetic nanoparticles	8 ng/L	Milk	[194]

Ochratoxin A	Colorimetric	—	20 nM	—	[195]

Ochratoxin A	Electrochemical impedance	Graphene oxide, gold nanoparticles	0.74 pM	Red wine	[196]

Ochratoxin A	Fluorescence	—	1 ng/mL	Beer	[197]

Ochratoxin A	Electrochemical redox current	Gold nanoparticles	0.75 pM	Red wine	[198]

Ochratoxin A	Electrochemiluminescence	Loop-mediated isothermal amplification	10 fM	Red wine	[199]

Ochratoxin A	Fluorescence	—	2 pg/mL	Wheat	[200]

Ochratoxin A	Localized surface plasmon resonance	—	1 nM	Corn powder	[201]

Ochratoxin A	RT-qPCR	—	1 fg/mL	Red wine	[202]

Ochratoxin A	Fluorescence	—	0.2 ng/mL	Red wine	[203]

Ochratoxin A	Chemiluminescence	Nicking endonuclease	0.3 pg/mL	Wheat	[204]

Ochratoxin A	Electrochemical impedance	—	0.25 ng/mL	Beer	[205]

Ochratoxin A	Electrochemical redox current	—	0.1 ng/mL	Beer	[206]

Ochratoxin A	Electrochemical impedance	—	0.12 ng/mL	Beer	[207]

Ochratoxin A	Electrochemical differential pulse voltammetry	Horseradish peroxidase, alkaline phosphatase, superparamagnetic nanoparticles	0.15 ng/mL	Red wine	[208]

Ochratoxin A	Electrochemical flow-based	—	0.05 *µ*g/L	Beer	[209]

Ochratoxin A	Colorimetric lateral flow	Gold nanoparticles	1 ng/mL, 0.18 ng/mL	Red wine	[210]

Ochratoxin A	Fluorescence lateral flow	Quantum dots	1.9 ng/mL	Red wine	[211]

Ochratoxin A	Electrochemical electric current	Horseradish peroxidase	0.07 ± 0.01 ng/mL	Wheat	[212]

Ochratoxin A	Electrochemical redox current	Gold nanoparticles	30 pg/mL	Red wine	[213]

Ochratoxin A	Electrochemical impedance	Horseradish peroxidase	0.4 pg/mL	Wheat	[214]

Ochratoxin A	Electrochemical redox current	—	0.095 pg/mL	Red wine	[215]

Ochratoxin A	Chemiluminescence	Upconversion nanoparticles	0.1 pg/mL	Maize	[216]

Ochratoxin A	Electrochemical impedance	Silver nanoparticles	0.05 nM	Beer	[217]

Ochratoxin A	Electrochemical impedance	Loop-mediated isothermal	0.3 pM	Red wine	[218]

Ochratoxin A	Fluorescence	—	0.8 ng/mL	Corn	[219]

Ochratoxin A	Fluorescence	Single-stranded signal probes	20 pg/mL	Wheat	[220]

Ochratoxin A	Fluorescence	Terbium	0.08–5.42 ng/mL	Wheat	[221]

Ochratoxin A	Electrochemical impedance	Redox probe	0.12–0.4 nM	Coffee, flour, wine	[222]

Ochratoxin A	Fluorescence resonance energy transfer	—	2 pg/mL	Maize flour	[223]

Ochratoxin A	Electrochemical redox current	Exonuclease digestion	1.0 pg/mL	Wheat starch	[224]

Ochratoxin A	Electrochemical electric current	Rolling circle amplification	0.2 pg/mL	Red wine	[225]

Ochratoxin A	Fluorescence	—	21.8 nM	Red wine	[226]

Ochratoxin A	Electrochemiluminescence	Gold nanoparticles	0.007 ng/mL	Wheat	[227]

Ochratoxin A	Colorimetric	—	2.5 nM	Red wine	[228]

Ochratoxin A	Colorimetric	—	4 nM	Red wine	[229]

Ochratoxin A	Fluorescence	—	24.1 nM	Beer	[230]

Ochratoxin A	Electrochemical impedance	—	0.1 ng/mL	—	[231]

Ochratoxin A	Fluorescence	—	0.01 ng/mL	Maize flour	[232]

**Table 8:** Summary table of ssDNA MREs based biosensors for the detection of chemical toxins.

Target	Detection methods	Enhancers	LOD	Relevant sample	Reference
Bisphenol A	Fiber optic fluorescence	—	1.86 nM	Waste water	[233]

Bisphenol A	Resonance light scattering	Gold nanoparticles	0.012–0.28 ng/mL	Supermarket ticket	[234]

Bisphenol A	Electrochemical redox current	Gold nanoparticles dotted graphene	5 nM	Milk	[235]

Bisphenol A	Colorimetric/fluorescent	—	0.1 ng/mL 0.01 pg/mL	Water	[236]

Bisphenol A	Colorimetric	—	0.1 ng/mL	Tap water	[237]

Bisphenol A	Colorimetric lateral flow	Gold nanoparticles	76 pg/mL	Tap water	[238]

Bisphenol A	Resonance Rayleigh scattering	Gold nanoparticles	83 pg/mL	Tap water	[239]

Bisphenol A	Electrochemical electric current	Single-walled carbon nanotubes field effect transistor	10 fM to 1 pM		[240]

Bisphenol A	Plasmonic chirality	—	8 pg/mL	Tap water	[241]

17*β*-estradiol (E2)	Photoelectrochemical	Titanium oxide nanotubes arrays	33 fM	Medical waste water, lake water, and tap water	[242]

17*β*-estradiol (E2)	Electrochemical impedance	—	2.0 pM	Human urine	[243]

17*β*-estradiol (E2)	Fluorescence	—	2.1 nM	Waste water	[244]

Acetamiprid	Colorimetric	—	5 nM	Soil	[245]

Acetamiprid	Electrochemical impedance	Gold nanoparticles	1 nM	Waste water, tomatoes	[246]

4 organophosphorus pesticides phorate, profenofos, isocarbophos, and omethoate	Fluorescence polarization	—	19.2, 13.4, 17.2, 23.4 nM	Chinese cabbage	[247]
